# Whole genome analysis of *Bacillus velezensis* YNK-FB0059 and its multifunctional plant growth-promoting and biocontrol potential

**DOI:** 10.3389/fmicb.2026.1748090

**Published:** 2026-01-30

**Authors:** Ende Liu, Yan Chen, Yuchun Yao, Weihua Pei, Te Pu, Zhufeng Shi, Yanru Cao, Peiwen Yang

**Affiliations:** 1 College of Agronomy and Life Sciences, Kunming University, Kunming, China; 2 Institute of Agricultural Environment and Resources, Yunnan Academy of Agricultural Sciences, Kunming, China

**Keywords:** *Bacillus velezensis*, biological control, plant growth promotion, rhizosphere probiotic, secondary metabolites, whole-genome analysis

## Abstract

**Introduction:**

This study conducted a systematic investigation of *Bacillus velezensis* YNK-FB0059 from genome to phenotype, aiming to comprehensively elucidate its genetic basis and functional traits through whole-genome sequencing and multi-dimensional *in vitro* validation experiments, thereby revealing its great potential as a multifunctional agricultural microorganism.

**Methods:**

Whole-genome sequencing combined with multi-platform bioinformatics analysis was employed to systematically mine secondary metabolite biosynthesis gene clusters, plant growth-promoting genes, and environmental adaptability genes of YNK-FB0059. The complete genome sequence of *B. velezensis* YNK-FB0059 has been deposited in the GenBank database under the accession number CP140613.1. Targeted experiments were designed and conducted, including plate confrontation assays against pathogenic microorganisms, spore germination and mycelial growth inhibition tests, assessments of phosphorus and potassium solubilization and nitrogen fixation capabilities, detection of siderophore and IAA secretion, biofilm formation analysis, and seed germination and pot-based growth promotion experiments.

**Results:**

Genomic analysis revealed that YNK-FB0059 has a chromosome size of 4.02 Mb, containing 14 secondary metabolite biosynthesis gene clusters encoding various antimicrobial substances such as surfactin, fengycin, bacilysin, and macrolactin H. Complete plant immunity activation gene systems (e.g., flagellin, chemotaxis proteins) and multiple growth-promoting pathways (e.g., nitrogen and sulfur metabolism, tryptophan synthesis) were also identified. *In vitro* experiments demonstrated that YNK-FB0059 exhibited broad-spectrum antifungal activity against eight important plant pathogenic fungi (inhibition rate: 77.8–88.6%). Its fermentation broth significantly inhibited pathogen spore germination, with a 24-h inhibition rate of 68.35%, and caused mycelial deformation and breakage. Additionally, YNK-FB0059 showed efficient phosphorus and potassium solubilization, nitrogen fixation, siderophore production, and IAA secretion (42.55 μg·mL^−1^ after 48 h). Biofilm formation reached 148 mg, and it significantly promoted seed germination and seedling growth in crops such as tomato and rapeseed.

**Discussion:**

Phylogenetic analysis combined with ANI/dDDH values confirmed its identity as *B. velezensis*. *B. velezensis* YNK-FB0059 exhibits excellent integrated traits of biocontrol, growth promotion, and rhizosphere colonization. Its rich secondary metabolite blueprint and complete genetic foundation for plant interaction make it an ideal candidate for developing efficient biopesticides and biofertilizers, holding significant application value in sustainable agricultural development.

## 1 Introduction

The continuously growing global population presents severe challenges to global food security, while modern agriculture heavily relies on chemical fertilizers and pesticides to maintain high yields (Liu et al., 2014). However, this production model has inevitably led to a series of environmental and health problems, including soil degradation, water pollution, increased pest and disease resistance, and agricultural product residues (Zhang et al., 2023). Consequently, developing and promoting environmentally friendly and sustainable agricultural technologies has become an urgent priority. Among these, the utilization of Plant Growth-Promoting Rhizobacteria (PGPR) as an effective biological strategy has shown great potential in promoting plant growth, controlling soil-borne diseases, and enhancing crop stress resistance, offering a viable pathway to reduce the use of chemical inputs (Gong et al., 2018).

*Bacillus* species are considered one of the most promising groups of plant growth-promoting bacteria due to their ability to form highly resistant spores, facilitating industrial production, storage, and application (Cai et al., 2018). Among them, *Bacillus velezensis* has rapidly become a research hotspot in the field of agricultural microbiology since its reclassification and establishment as a distinct species (Lim et al., 2018). Numerous studies have demonstrated that *Bacillus velezensis* possesses multiple beneficial functions. Primarily, it can produce a rich array of antimicrobial secondary metabolites, such as lipopeptides (e.g., surfactin, iturin, fengycin) and polyketides (e.g., difficidin, macrolactin), exhibiting significant antagonistic effects against a wide range of plant pathogenic fungi and bacteria, making it a broad-spectrum biocontrol agent (Li and Li, 2015). Secondly, it can promote nutrient transformation and uptake through nitrogen fixation, phosphate solubilization, and siderophore secretion, and directly stimulate plant growth and root development by synthesizing phytohormones such as Indole-3-acetic acid (IAA) (Zhong et al., 2022). Furthermore, it can induce systemic resistance in plants, thereby enhancing their tolerance to both biotic and abiotic stresses (Chowdhury et al., 2023). For instance, whole-genome analysis of the model strain *B. velezensis* FZB42 revealed that over 10% of its genome is dedicated to encoding antibiotics and secondary metabolites, providing a genetic explanation for its potent biocontrol and growth-promoting capabilities (Zhang et al., 2020).

Although numerous studies have reported the probiotic effects of *B. velezensis*, it is crucial to recognize that its functionality exhibits significant strain-specificity (Fan et al., 2017). Different strains, due to subtle genetic variations, may possess distinct metabolic profiles and ecological functions. Therefore, evaluating a strain’s potential based solely on phenotypic experiments is insufficient. With the rapid advancement of sequencing technologies, whole-genome sequencing provides a powerful tool for gaining in-depth understanding of strain functions (Xiong and Wang, 2025). Through genomic analysis, we can systematically predict a strain’s potential metabolic capabilities, biocontrol mechanisms, and growth-promoting traits, thereby explaining its phenotype at the genotypic level and achieving a leap from “what” to “why” (Zhang et al., 2019). This research strategy, linking genotype to phenotype, not only provides clear theoretical guidance for *in vitro* and field validation experiments but also lays a solid foundation for subsequent strain patent protection, genetic improvement, and the development of efficient microbial inoculants (Qin et al., 2011).

This study focuses on *B. velezensis* YNK-FB0059, a strain isolated from the rhizosphere of a healthy crop. Preliminary experiments indicated that this strain exhibits significant antagonistic activity against various plant pathogens and effectively promotes seed germination and tomato seedling growth. However, the underlying molecular mechanisms remain unclear. Based on this, our research aims to systematically analyze the genomic characteristics of strain YNK-FB0059 through whole-genome sequencing and in-depth bioinformatics mining, with a focus on predicting gene clusters related to secondary metabolite synthesis, phytohormone production, and nutrient transformation. Furthermore, we will validate its biocontrol and growth-promoting functions through *in vitro* antagonism assays, seed germination tests, and pot experiments. This study aims to comprehensively elucidate the functional potential and genetic basis of *B. velezensis* YNK-FB0059 from both genomic and phenotypic perspectives, providing a scientific basis for its development and application as a novel multifunctional microbial inoculant.

## 2 Materials and methods

### 2.1 Culture media

To ensure the reproducibility of the experiments, all culture media and related reagents used in this study were purchased from Qingdao Haibo Biotechnology Co., Ltd. (Qingdao, China). The specific formulations of each medium are described as follows: Nutrient Agar (NA) medium contains 10 g·L^−1^ peptone, 3 g·L^−1^ beef extract, 5 g·L^−1^ sodium chloride, and 18 g·L^−1^ agar with the pH adjusted to 7.0; Nutrient Broth (NB) medium has the same formulation as NA medium except without the addition of agar, and its pH is 7.0; Potato Dextrose Broth (PDB) medium consists of 200 g·L^−1^ potato and 20 g·L^−1^ glucose with a pH of 7.0; Potato Dextrose Agar (PDA) medium is formulated based on PDB medium with 15 ~ 20 g·L^−1^ agar added, and the pH is 7.0; Luria-Bertani(LB) medium contains 10 g·L^−1^ tryptone, 5 g·L^−1^ yeast extract, and 10 g·L^−1^ sodium chloride; MSgg medium includes 100 mmol/L 3-(N-morpholino)propanesulfonic acid (MOPS), 5 mmol/L potassium phosphate, 2 mmol/L magnesium chloride, 700 μmol/L calcium chloride, 50 μmol/L manganese chloride, 50 μmol/L iron chloride, 1 μmol/L zinc chloride, 2 μmol/L thiamine, 0.5% (mass/volume ratio) glycerol, 0.5% (mass/volume ratio) glutamic acid, 50 μg·mL^−1^ tryptophan, 50 μg·mL^−1^ phenylalanine, and 50 μg·mL^−1^ threonine, with the pH adjusted to 7.0.

### 2.2 Tested strain

*Bacillus velezensis* strain YNK-FB0059 was isolated from soil samples collected in the Wuliang Mountain National Nature Reserve, Dali Bai Autonomous Prefecture, Yunnan Province, China. Soil samples were collected from five sampling plots, with 10 sampling points per plot. At each point, five subsamples were combined to form a composite sample, yielding a total of 50 soil samples, which were stored in sterile 50 mL centrifuge tubes and transported to the laboratory for bacterial isolation.

For bacterial isolation, 2 g of homogenized soil was suspended in 200 mL of sterile water, supplemented with glass beads, and shaken at 28 °C for 4–6 h. Aliquots (200 μL) of the resulting suspension were evenly spread on Nutrient Agar (NA) plates and incubated at 28 °C for 24 h. Morphologically distinct colonies were selected as candidate strains for further study.

For molecular identification, each candidate strain was inoculated into Nutrient Broth (NB) and cultured at 30 °C with shaking at 180 rpm until the OD_600_ reached approximately 1 (≈1 × 10^9 CFU/mL). Genomic DNA was extracted using the TaKaRa Mini BEST Bacteria Genomic DNA Extraction Kit Ver.3.0 according to the manufacturer’s instructions. The 16S rRNA gene and housekeeping genes gyrA and rpoB were amplified by PCR using universal primers (16S rRNA: 27F/1492R; gyrA: F/R; rpoB: 2292F/3354R). PCR products were purified and sequenced. Sequence data were compared against the GenBank database using BLAST to determine species identity. The sequences showing the highest similarity were used as references for multiple sequence alignment with ClustalX. Phylogenetic relationships between YNK-FB0059 and reference *Bacillus* strains were inferred using the Neighbor-Joining method in MEGA 7.0 with 1,000 bootstrap replicates.

The strain YNK-FB0059 was maintained at the Institute of Agricultural Environment and Resources, Yunnan Academy of Agricultural Sciences. Preliminary characterization indicated typical *Bacillus* morphology and Gram-positive rod-shaped cells capable of forming endospores. This strain was used in all subsequent functional and genomic analyses in the present study.

The tested pathogenic microorganisms involved in this study refer to a set of plant pathogenic microbes selected to evaluate the antagonistic activity of *B. velezensis* strain YNK-FB0059. The indicator pathogens involved in this study included *Fusarium acuminatum*, *Fusarium oxysporum* f. sp. *cubense* (banana Fusarium wilt pathogen), *Fusarium solani*, *Fusarium graminearum*, *Fusarium equiseti*, *Phoma matteuciicola* (leaf spot pathogen of *Amomum tsaoko*), *Diaporthe eres* (branch blight pathogen of *Amomum tsaoko*), and *Phytophthora nicotianae*. All pathogens were also isolated, identified, and maintained by the same institute.

### 2.3 Strain and genome sequencing

The Whole Genome Shotgun (WGS) strategy was employed. Libraries with different insert sizes were constructed and sequenced using both second-generation sequencing (NGS) on the Illumina NovaSeq platform (2 × 150 bp paired-end reads) and third-generation single-molecule sequencing on the PacBio Sequel platform. Following sequencing, assembly was performed using software such as HiFiasm, Unicycler, and Flye. Additionally, the high-quality NGS data was used to polish the third-generation contigs with Pilon software, ultimately yielding a complete genome sequence (Ghelardi et al., 2012).

### 2.4 Genome annotation and analysis

Following genome assembly, repetitive sequences were predicted *de novo* using RepeatModeler (version 1.0.8) and identified/masked using RepeatMasker (version 4.0.5). For non-coding RNA analysis, tRNAs were predicted using tRNAscan-SE (Mordini et al., 2013) and rRNAs were predicted using Barrnap. Other non-coding RNAs were identified by comparison with the Rfam database (Li et al., 2023). Protein-coding genes (CDSs) across the entire genome were predicted using GeneMarkS (Ciftci et al., 2019). Prophages within the genome were predicted using PhiSpy (Milton and Cavanagh, 2023), and genomic islands were identified using IslandViewer 4 (Musik et al., 2021). The predicted protein-coding genes were functionally annotated by searching against databases including NCBI nr, eggNOG, KEGG, Swiss-Prot, GO, TCDB, Pfam, CAZy, and CARD. Secreted proteins and transmembrane proteins were predicted using SignalP and TMHMM, respectively.

### 2.5 Prediction of NP-BGCs in the genome of strain YNK-FB0059

We utilized an integrated approach employing antiSMASH 6 (Wei et al., 2023) in combination with ClusterBlast, Active Site Finder, Cluster Pfam analysis, Subcluster Blast, and PRISM 4 to identify and compare the Biosynthetic Gene Clusters (BGCs) of secondary metabolites in the *B. velezensis* YNK-FB0059 genome. Additionally, BAGEL 4 (Wei et al., 2023) was employed to mine RiPPs (Ribosomally synthesized and post-translationally modified peptides) and bacteriocins within the BGCs, while PRISM 4 was used to predict the structures of secondary metabolites produced by the strain (Hofmann et al., 2000). These database systems incorporated principles including Hidden Markov Models (HMM), the BLAST algorithm, and utilized databases such as Pfam, GenBank, UniProt KB, Bactibase, CAMP(R3), and MIBiG for BGC annotation. Furthermore, NapDos was utilized to search for KS (ketosynthase) and C (condensation) domains within these genomic sequences (Wang et al., 2020).

### 2.6 Analysis of genes in strain YNK-FB0059 associated with plant growth promotion and immunity enhancement

Functional annotation of the genes encoded by the strain was performed based on search results from databases including NCBI nr, eggNOG, KEGG, Swiss-Prot, GO, Pfam, CAZy, and CARD. Furthermore, based on the database annotation results, genes related to plant growth promotion and plant immunity enhancement were systematically screened within the genome annotation results of strain YNK-FB0059.

### 2.7 *In vitro* antifungal activity

Using the aforementioned eight indicator pathogens as test organisms, the *in vitro* antifungal activity of *Bacillus velezensis* strain YNK-FB0059 was evaluated using the dual culture method. Briefly, a 5 mm diametermycelial plug of each pathogen was inoculated at the center of a PDA plate. Subsequently, *B. velezensis* strain YNK-FB0059 was inoculated 25 mm away from the pathogen mycelial plug in a cross-streak pattern. Each treatment was performed with three biological replicates. All plates were incubated in the dark at 25–28 °C in a constant temperature incubator for 5–7 days. The colony diameter of each group was measured, and the inhibition rate was calculated according to the following formula:


$$\text{Inhibition rate}(\%)=(D_{\mathrm{ck}}-D_{\mathrm{tr}})/(D_{\mathrm{ck}}-5)\times 100$$

Where *D_ck_* represents the average colony diameter of the control group, defined as the mean diameter of pathogen colonies grown on PDA plates inoculated only with the pathogen (without *B. velezensis* YNK-FB0059) after incubation (unit: mm), and *D_tr_* represents the average colony diameter of the treatment group, defined as the mean diameter of pathogen colonies grown on PDA plates co-inoculated with both the pathogen and *B. velezensis* YNK-FB0059 (unit: mm). The value of 5 mm corresponds to the diameter of the initial pathogen mycelial plug and was introduced to eliminate the influence of the initial inoculum size on colony growth measurements, thereby ensuring that the calculated inhibition rate accurately reflects the inhibitory effect of the test strain on pathogen mycelial growth.

For colony diameter determination, a cross-measurement method was adopted: two perpendicular diameters of each colony were measured, and the average of the two values was taken as the colony diameter of a single plate. For both the control and treatment groups, the mean colony diameter calculated from three replicate plates was used as the final *D_ck_* or *D_tr_,* ensuring the accuracy and representativeness of the experimental data.

The calculation procedure was as follows: first, the net growth diameter of the control group was obtained by subtracting 5 mm from Dck (Dck-5), and the net growth diameter of the treatment group was calculated as Dtr-5. The inhibition rate was then derived by dividing the growth difference between the control and treatment groups by the net growth diameter of the control group and multiplying the result by 100. A higher inhibition rate indicates stronger antifungal activity of the test strain.

### 2.8 Effect of *Bacillus velezensis* YNK-FB0059 on pathogen mycelium

Based on the dual-culture plate confrontation assay described in Section 2.7, *Bacillus velezensis*ynk-FB0059 and the *Phoma* leaf spot pathogen of *Amomum tsaoko* were co-cultured on PDA plates at a constant temperature of 25 °C. Each treatment was replicated three times. After 15 days of incubation, mycelia were collected from the margin of the inhibition zone at the pathogen–bacterium interaction area and examined using scanning electron microscopy (SEM).

### 2.9 Effect of the *Bacillus velezensis* YNK-FB0059 on the conidial germination of the *Phoma* leaf spot pathogen of *Amomum tsaoko*

The *Phoma* leaf spot pathogen was cultured on PDA medium for 15 days. An appropriate amount of sterile water was then added, and conidia were scraped off the surface using a sterile pipette tip. The suspension was filtered through sterile gauze to obtain a pathogenic spore suspension. *Bacillus velezensis* YNK-FB0059 was cultured at 30 °C with shaking at 180 rpm for 24 h. The optimal routine cultivation temperature for strain YNK-FB0059 is 30 °C, ensuring proper bacterial growth and metabolic activity. This temperature was used for preparing the culture filtrate to maintain stability and activity of bacterial secreted metabolites. The culture was then centrifuged at 4 °C and 8,000 rpm for 5 min. The supernatant was collected and passed through a 0.22 μm sterile filter to remove bacterial cells, resulting in a sterile cell-free culture filtrate. This filtrate was mixed 1:1 (v/v) with the pathogen spore suspension to prepare the test mixture. A control was prepared by mixing the pathogen spore suspension with an equal volume of sterile water (instead of filtrate). The mixtures were incubated at 25 °C, a temperature chosen to simulate natural growth conditions of the pathogen and avoid inhibition of spore germination by higher temperatures, allowing more accurate assessment of the inhibitory effect of the bacterial strain. The number of germinated spores was recorded at 4, 14, and 24 h post-inoculation.

Spore germination rate and germination inhibition parameters were evaluated according to the following formulas:


$$\begin{array}{l} \text{Spore Germination Rate}\;(\%)=\\ (\text{Number of Germinated Spores}/\text{Total Number of Spores})\times 100 \end{array}$$

$$\begin{array}{l} (\text{Spore Germination Rate of Control Group}-\\ \text{Spore Germination Rate of Treatment Group})/\\ (\text{Spore Germination Rate of Control Group})\times 100 \end{array}$$

### 2.10 Morphological and molecular biological identification of *Bacillus velezensis* YNK-FB0059

The morphological identification and physiological-biochemical characterization of strain YNK-FB0059 were performed according to the methods described in Bergey’s Manual of Systematic Bacteriology (Buchanan and Gibbons, 1984) and Manual for the Identification of Common Bacterial Systematics (Dong and Cai, 2001; Leiman et al., 2014). Furthermore, based on the whole-genome sequencing data, a phylogenetic tree was constructed using the Neighbor-joining method in MEGA 7.0 software to illustrate the evolutionary relationships between the tested strain and reference strains. The Bootstrap value was set to 1,000 replicates, and other parameters were set to default. Additionally, differences between YNK-FB0059 and various *Bacillus* reference genomes were further determined based on Average Nucleotide Identity (ANI) and digital DNA–DNA Hybridization (dDDH) analyses.

### 2.11 Determination of sulfur oxidation, phosphorus solubilization, potassium release, nitrogen fixation, and siderophore production by *Bacillus velezensis* YNK-FB0059

Plate-based qualitative assays were employed to evaluate the plant growth–promoting (PGP) traits of *Bacillus velezensis* YNK-FB0059, including phosphate solubilization, potassium release, nitrogen fixation, sulfur oxidation, and siderophore production. For phosphate solubilization, potassium release, nitrogen fixation, and sulfur oxidation, the strain was spot-inoculated onto the corresponding selective media and incubated at 30 °C for 48 h. The formation of a clear halo zone or characteristic color change around the colonies was considered indicative of positive activity for the respective function.

Phosphate solubilization was assessed using a selective medium containing lecithin as an organic phosphorus source. The medium consisted of (per liter): ammonium sulfate (0.5 g), yeast extract (0.5 g), NaCl (0.3 g), KCl (0.3 g), MgSO₄ (0.3 g), FeSO₄ (0.03 g), MnSO₄ (0.03 g), lecithin (0.2 g), CaCO₃ (1.0 g), and agar (20 g), with the pH adjusted to 7.0–7.5. The appearance of a distinct clear halo surrounding the colony was regarded as a positive result for organic phosphorus solubilization (mineralization).

Potassium release was evaluated using a selective medium supplemented with potassium feldspar as the insoluble potassium source. The medium contained (per liter): sucrose (10 g), Na₂HPO₄ (1 g), (NH₄)₂SO₄ (0.5 g), MgSO₄·7H₂O (1 g), yeast extract (0.2 g), NaCl (0.1 g), CaCO₃ (0.1 g), FeCl₃ (0.005 g), potassium feldspar powder (5 g; washed 4–5 times with distilled water prior to use), and agar (20 g). The formation of a transparent halo around the colony indicated potassium-releasing activity.

Nitrogen fixation was determined using a nitrogen-free selective medium composed of (per liter): glucose (10 g), Na₂HPO₄ (0.2 g), NaCl (0.2 g), MgSO₄·H₂O (0.2 g), K₂SO₄ (0.2 g), CaCO₃ (5 g), and agar (20 g). The formation of a clear halo zone surrounding the colony was considered indicative of nitrogen-fixing activity.

Sulfur oxidation was assessed using MST selective medium, which consisted of (per liter): K₂HPO₄ (0.1 g), NaHCO₃ (0.2 g), NH₄Cl (0.1 g), glucose (5.0 g), and yeast extract (5.0 g), with bromocresol purple (0.008 g) added as a pH indicator. The pH was adjusted to 7.9–8.1. After sterilization and cooling to 40–50 °C, Na₂S₂O₃ (5.0 g) was added. The appearance of a yellow halo zone surrounding the colony was considered a positive sulfur oxidation reaction, as sulfur-oxidizing bacteria convert thiosulfate into sulfate, producing acidic metabolites that lower the local pH and cause the bromocresol purple to turn yellow.

Siderophore production was evaluated using the Chrome Azurol S (CAS) agar assay. The CAS medium consisted of (per liter): Chrome Azurol S (60.5 mg), hexadecyltrimethylammonium bromide (HDTMA; 72.9 mg), FeCl₃·6H₂O (2.645 mg), Na₂HPO₄·2H₂O (295.25 mg), Na₂HPO₄·12H₂O (1213.5 mg), NH₄Cl (125 mg), KH₂PO₄ (37.5 mg), NaCl (62.5 mg), and agar (20 g). The strain was inoculated at the center of CAS agar plates and incubated at 30 °C for 48 h, with uninoculated plates serving as controls. All experiments were conducted in triplicate. A color change of the medium from blue to orange or yellow indicated siderophore production, as described previously (He et al., 2020).

### 2.12 Determination of IAA secretion by *Bacillus velezensis* YNK-FB0059

An IAA standard (10 mg, ≥98% purity, Shanghai YuanYe Bio-Technology Co., Ltd., China) was dissolved in a small volume of ethanol and the solution was brought to a final volume of 100 mL with distilled water to achieve a stock concentration of 100 μg·mL^−1^. This stock solution was then serially diluted to prepare standard solutions with concentrations of 0, 10, 20, 30, 40, 50, and 60 μg·mL^−1^. For each standard, 4 mL was mixed with an equal volume of color development reagent. The mixtures were incubated in the dark at 40 °C for 40 min to allow color development, after which the OD_535_ was measured. A standard curve was plotted using these measurements, with the equation y = 0.0069x + 0.0058 and a correlation coefficient *R*^2^ = 0.9989. For the experimental samples, the bacterial suspension and a blank control were centrifuged at 10,000 rpm for 10 min. Subsequently, 4 mL of each supernatant was mixed 1:1 (v/v) with the color development reagent. The mixtures were allowed to stand in the dark for 40 min. The OD_535_ was measured using a spectrophotometer, with the blank color development reagent used for zero adjustment. This procedure was performed in triplicate. Measurements were taken over 7 consecutive days to monitor color changes at different fermentation times. The IAA concentration produced by the strain at each time point was calculated by comparison with the standard curve, allowing determination of the optimal fermentation time for maximum IAA production (Prasanth and Mathivanan, 2010).

### 2.13 Determination of biofilm formation capacity of *Bacillus velezensis* YNK-FB0059

Biofilm formation is a key factor determining the colonization ability of functional strains on plant roots. The biofilm-forming ability of *Bacillus velezensis* functional strain YNK-FB0059 was evaluated under static conditions using MSgg medium, a nutrient-poor liquid medium widely used to induce structured biofilm formation in *Bacillus* species (Branda et al., 2001; Leiman et al., 2014) In brief, strain YNK-FB0059 was cultured in LB medium until the OD < sub > 600</sub > reached 1.0, then cells were harvested by centrifugation at 8,000 rpm, washed three times with sterile water, and resuspended in an equal volume of MSgg medium. For qualitative analysis, 10 μL of the cell suspension was inoculated into each well of a 48-well plate (1 mL MSgg medium per well) and incubated statically at 30 °C for 16 h to assess biofilm formation based on colony morphology. For quantitative analysis, 100 μL of the cell suspension was inoculated into each well of a 6-well plate (10 mL MSgg medium per well) and incubated under the same conditions. After 16 h of incubation, biofilms formed in each well were collected by sterile membrane filtration and quantified by weighing. All treatments were performed in three independent biological replicates (Qiao et al., 2014).

### 2.14 Determination of seed germination-promoting ability of the strain

Four treatments were established: (1) Strain YNK-FB0059 was cultured in LB liquid medium in 500 mL Erlenmeyer flasks containing 300 mL medium, with an initial inoculum of 1% (v/v), and incubated at 30 °C on a rotary shaker at 180 rpm for 48 h. The optimal routine cultivation temperature for strain YNK-FB0059 is 30 °C, which ensures proper bacterial growth and metabolic activity. This temperature was used for preparing bacterial cells for seed treatment to maintain the stability and activity of secreted metabolites. The culture was then centrifuged at 4 °C and 8,000 rpm for 10 min. The supernatant was discarded, and the cell pellet was resuspended in sterile water to a final concentration of 1.0 × 10^8^ CFU·mL^−1^. (2) After fermentation for 48 h, the culture of strain YNK-FB0059 was centrifuged at 4 °C and 8,000 rpm for 10 min. The supernatant was collected and diluted to 10^−1^, 10^−2^, and 10^−3^. (3) Sterile water. (4) IAA standard solution (100 mg·L^−1^) (Xiong et al., 2020). Seeds were treated by soaking in the respective solutions for 24 h according to the above groups. Subsequently, seeds from each group were placed in 9 cm transparent Petri dishes lined with 2–3 layers of sterilized filter paper, with 15 seeds per dish and three replicates per treatment (45 seeds total). The dishes were incubated in an artificial climate chamber at 27 °C with a photoperiod of 16 h light and 8 h darkness. The incubation temperature of 27 °C was chosen because it is optimal for seed germination, simulating natural conditions while avoiding inhibition of germination by higher temperatures, allowing accurate assessment of the bacterial strain’s promoting effect. After 7 days of cultivation, the germination rate and whole plant length were measured.

### 2.15 Evaluation of plant growth-promoting effects of strain YNK-FB0059 in tomato pot experiment

A pot experiment was conducted under greenhouse conditions to evaluate the growth-promoting effects of functional strain YNK-FB0059 on tomato seedlings. Six treatments were established, including two control groups and four fermentation broth treatments: CK1 (sterile water), CK2 (sterile NB liquid medium), 0 (undiluted fermentation broth), 10^−1^ (10-fold diluted fermentation broth), 10^−2^ (100-fold diluted fermentation broth), and 10^−3^ (1000-fold diluted fermentation broth). Each treatment consisted of 10 replicates, with one seedling per replicate.

Strain YNK-FB0059 was inoculated into NB liquid medium and cultured under shaking conditions at 30 °C and 180 rpm to obtain the fermentation broth. Tomato seedlings were transplanted into pots and allowed to grow for 7 days before being subjected to a root drenching treatment. Each seedling received 200 mL of the corresponding treatment solution: CK1 received 200 mL sterile water, CK2 received 200 mL sterile NB liquid medium, and the other treatments received 200 mL of the respective fermentation broth or its dilutions.

The seedling substrate was a commercial soil matrix (Monor brand, produced by Zhonghe Agricultural Science and Technology Development Co., Ltd., Huai’an, Jiangsu, China). The pots used had a diameter of 14.7 cm, a height of 12.5 cm, and a bottom diameter of 10.5 cm. Tomato seedlings were transplanted to the greenhouse in late March, and the total growth period was 37 days. Watering was carried out every 3–5 days during the growth period.

After 30 days of root drenching treatment, the seedlings were sampled for measurement. The measured parameters included plant height (cm), stem diameter (cm), root length (cm), root fresh weight (g), above-ground fresh weight (g), and above-ground dry weight (g). All data are presented as mean ± standard deviation, and different letters indicate significant differences among treatments at *p* < 0.05.

### 2.16 Statistical analysis

All experiments were performed with at least three independent biological replicates.

Data are presented as mean ± standard error (SE). Statistical analyses were conducted using Origin software. Differences among treatments were analyzed by one-way analysis of variance (ANOVA), and differences were considered statistically significant at *p* < 0.05.

## 3 Results

### 3.1 Morphological, physiological, biochemical, and molecular identification of strain YNK-FB0059

**Figure 1:**
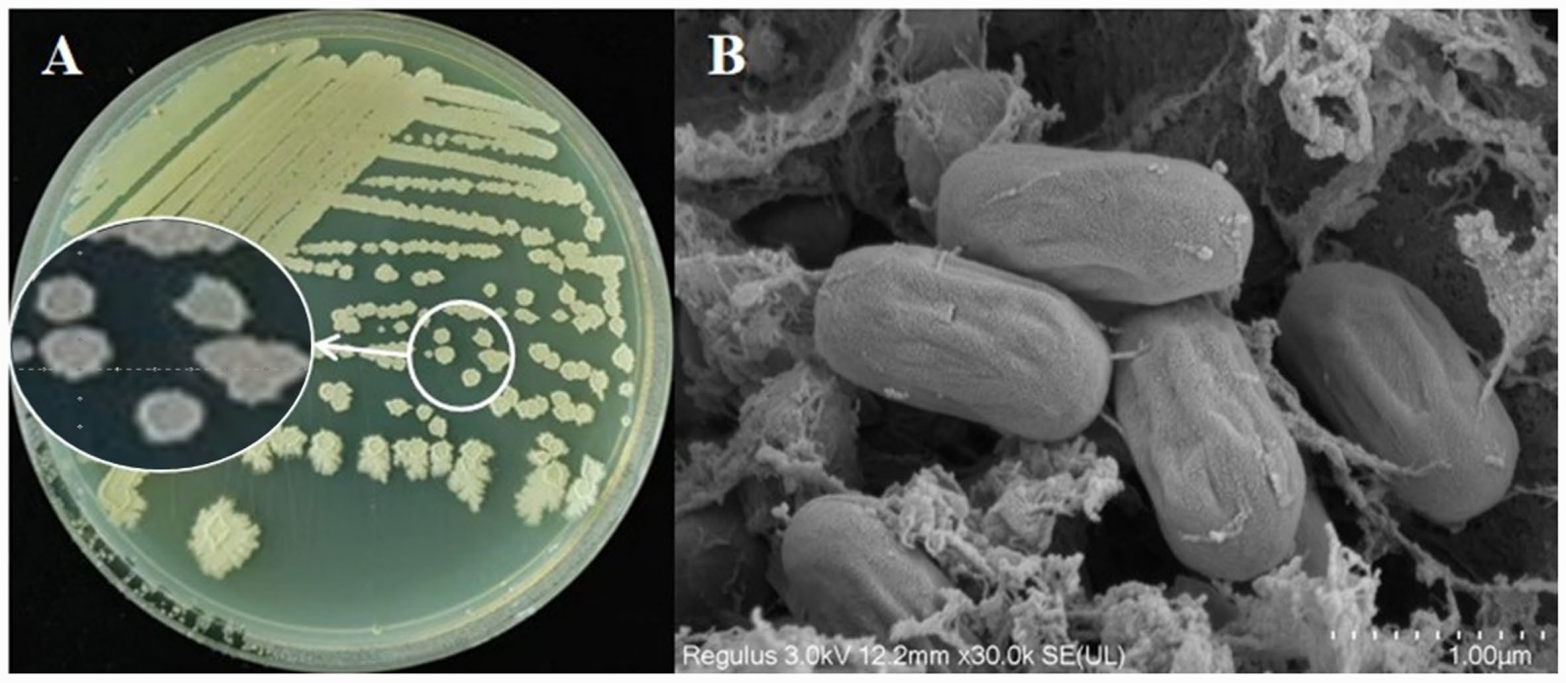
Morphological characterization of *B. velezensis* YNK-FB0059. **(A)** Colony morphology of YNK-FB0059 observed on nutrient agar. **(B)** Scanning electron microscopy image of rod-shaped YNK-FB0059 cells.

Strain YNK-FB0059 cells were rod-shaped and formed colonies with a creamy white center, lacking pigment production. The colonies exhibited irregular shapes, a viscous and wrinkled surface, and were slightly convex (Figures 1A,B). Physiological and biochemical characterization indicated that the strain was Gram-positive, aerobic, and capable of forming endospores. It tested positive for catalase, Voges-Proskauer (V-P) reaction, and citrate utilization. The strain also demonstrated the ability to produce indole, generate H₂S, and utilize mannitol and glucose. The minimum growth temperature was 4 °C, and it tolerated NaCl concentrations in the range of 0–12% (Table 1).

**Table 1:** Physiological and biochemical characteristics of *B. velezensis* YNK-FB0059.

Measuring item	No.
	YNK-FB0059
Gram staining	+
Form spores	+
Mycelial morphology	Rhabditiform
Catalase	+
V–P experiment	+
Methyl red	−
Gelatin liquefaction	+
Citrate	+
Minimum growth temperature	4°C
Growth salinity range	0–12%
Produce indoles	+
Produce H_2_S	+
Anaerobic growth	−
Mannitol	+
Glucose	+

“+” refers to positive reaction. “−” refers to negative reaction. All tests were repeated three times independently, and results were consistent across replicates.

**Figure 2:**
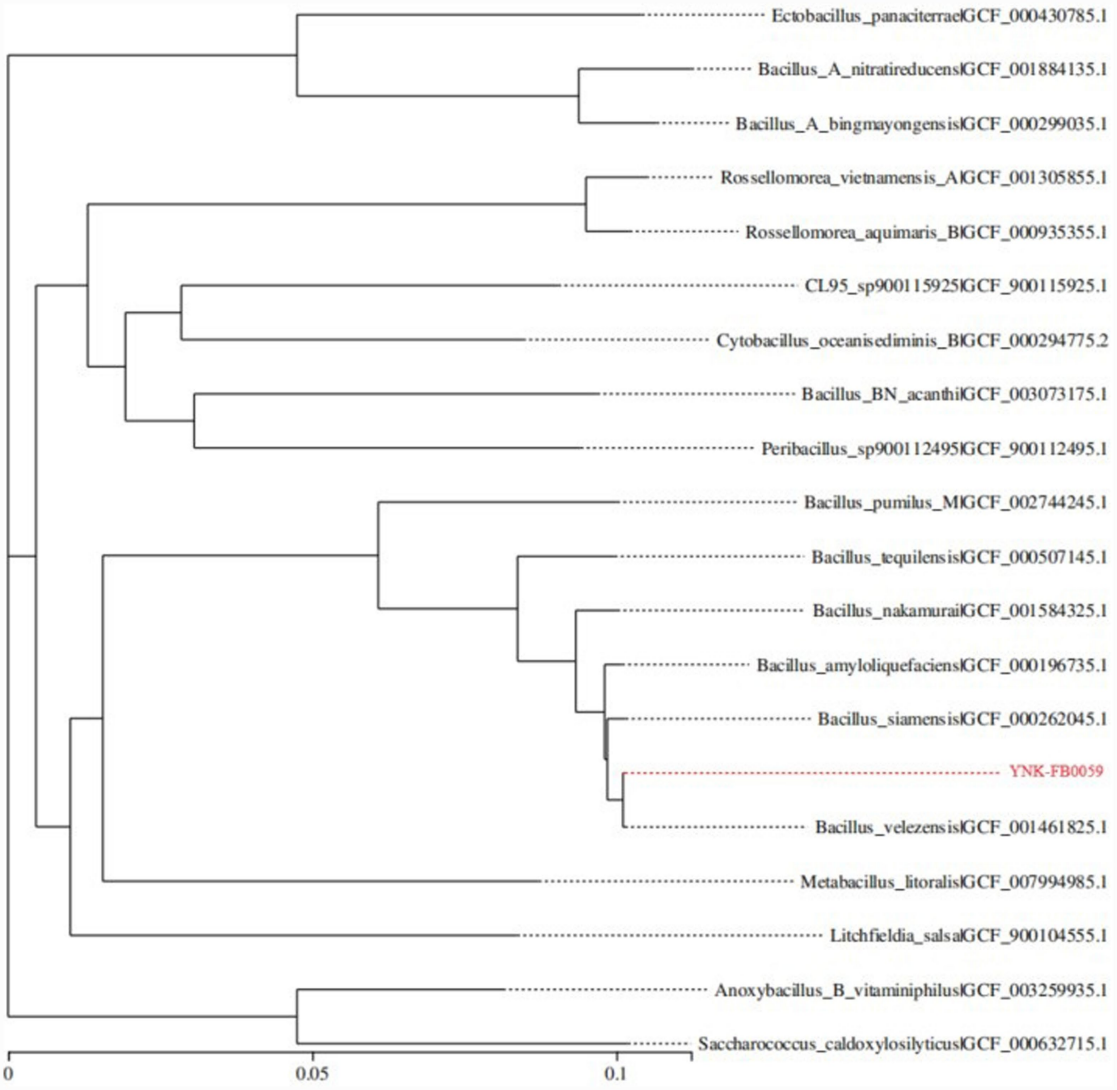
Phylogenetic tree of strain YNK-FB0059 based on 31 housekeeping genes.

Based on 31 housekeeping genes (*dnaG*, *frr*, *infC*, *nusA*, *pgk*, *pyrG*, *rplA*, *rplB*, *rplC*, *rplD*, *rplE*, *rplF*, *rplK*, *rplL*, *rplM*, *rplN*, *rplP*, *rplS*, *rplT*, *rpmA*, *rpoB*, *rpsB*, *rpsC*, *rpsE*, *rpsI*, *rpsJ*, *rpsK*, *rpsM*, *rpsS*, *smpB*, *tsf*), 19 of the most closely related strains at the species level were selected. A phylogenetic tree was constructed using the Neighbor-Joining (NJ) method in MEGA 6.0 software. The results showed that strain YNK-FB0059 clustered on the same branch as *B. velezensis* (GenBank accession no.: GCF_001461825.1) (Figure 2). Combined with the results of ANI and dDDH analyses (Table 2), as well as morphological, physiological, and biochemical identification, strain YNK-FB0059 was ultimately identified as *B. velezensis*.

**Table 2:** ANI and dDDH values of the YNK-FB0059 genome compared to the reference genomes of the genus *Bacillus*.

Reference genome	Accession number	ANI	DDH
*B. velezensis* FZB42	CP000560.2	98.37	92.6
*B. velezensis* SQR9	CP006890.1	98.36	91.1
*B. amyloliquefaciens* GKT04	CP072120.1	98.35	93.2
*B. velezensis* LS69	CP015911.1	98.31	93.3
*B. amyloliquefaciens* B408	CP101661.1	97.71	92.7
*B. siamensis* WYJ-E14	CP101610.1	94.52	85.8
*B. amyloliquefaciens* DSM 7	FN597644.1	94.09	80.1
*B. inaquosorum* KCTC 13429	CP029465.1	77.59	33.5
*B. subtilis* subsp. *spizizenii* ATCC 6633	CP034943.1	77.45	33.4
*B. atrophaeus globigii* BSS	CP007640.1	77.54	30.8
*B. mojavensis* UCMB5075	CP051464.1	77.44	32.7
*B. subtilis* TU-B-10	CP002905.1	77.37	33.5
*B. subtilis* 168	CP053102.1	77.32	32.2
*B. vallismortis* Bac111	CP033052.1	77.31	32.4
*B. halotolerans* ZB201702	CP029364.1	77.30	33.7
*B. stercoris* SMPL712	CP126678.1	77.13	34.3
*B. subtilis* NCIB 3610	CP020102.1	77.03	32.8
*B. paralicheniformis* Bac84	CP023665.1	72.96	15.9
*B. licheniformis* SCDB 14	CP014842.1	72.95	16.3
*B. sonorensis* SRCM101395	CP021920.1	72.84	16.1

### 3.2 Whole-genome sequencing analysis of *Bacillus velezensis* YNK-FB0059

To elucidate the genetic basis underlying the plant growth–promoting and biocontrol potential of *Bacillus velezensis* YNK-FB0059, whole-genome sequencing was performed, followed by comprehensive analyses of genomic features, functional annotation, and carbohydrate-active enzymes.

#### 3.2.1 Genome features and comparative analysis

**Figure 3:**
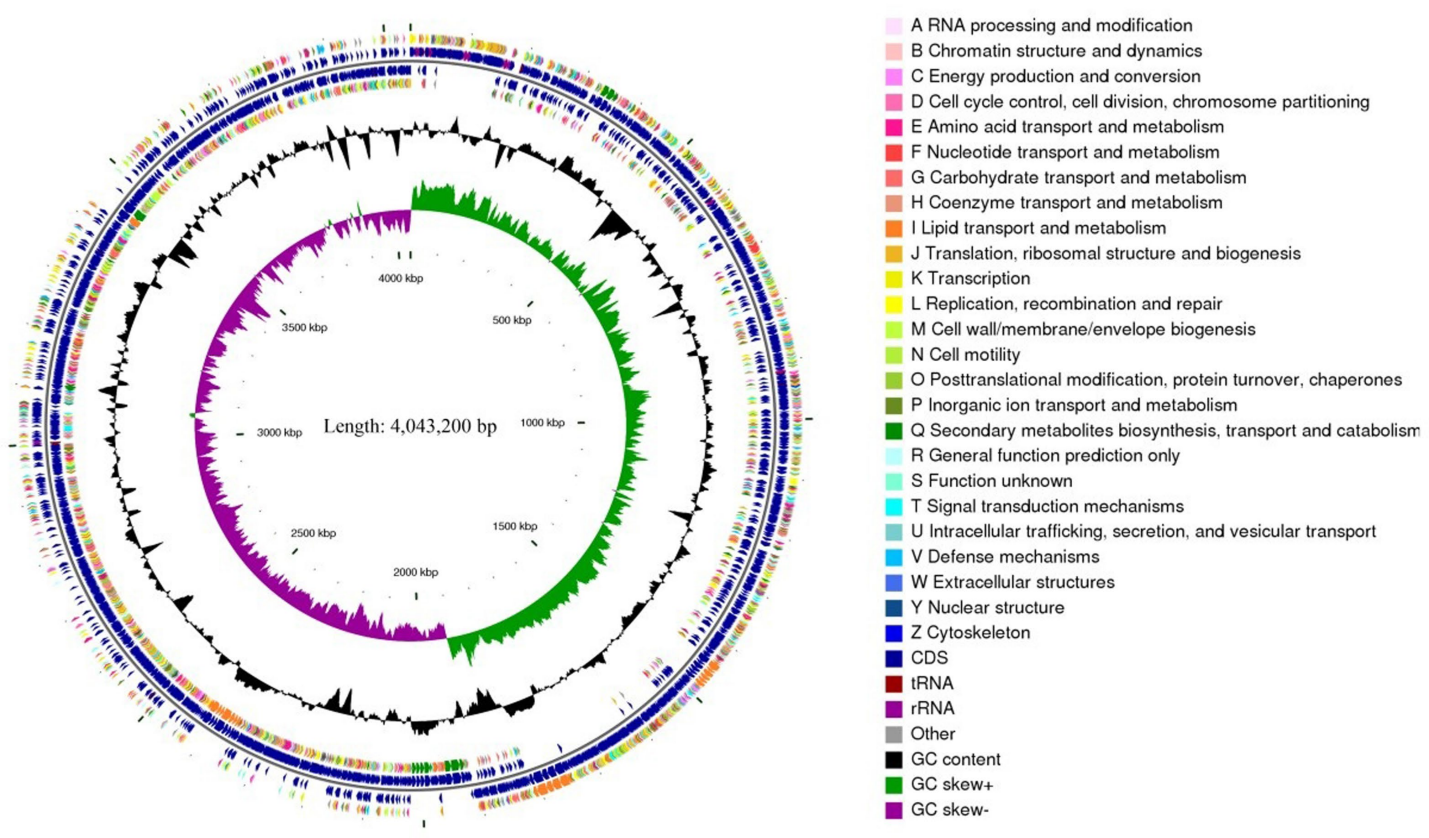
Genome circle map of *B. velezensis* YNK-FB0059. From the inside to the outside, the first circle represents the scale; the second lap represents GC skew; the third circle represents the GC content; the fourth and seventh circles represent the COG to which each CDS belongs; the fifth and sixth circles represent the position of CDS, tRNA, and RNA on the genome.

Whole-genome sequencing of *B. velezensis* YNK-FB0059 revealed (Table 3; Figure 3) a chromosome size of 4.02 Mb (4,043,200 bp) with a GC content of 46.39%. The genome encodes 3,848 genes, representing a coding density of 88.83%. The average length of the protein-coding genes is 931.75 bp. One plasmid was identified, containing 27 tandem repeats with a total length of 5,536 bp. The genome also contains 86 tRNA genes, 9 copies of 5S rRNA, 9 copies of 16S rRNA, and 9 copies of 23S rRNA (27 rRNA genes in total). Furthermore, comparative analysis of the *B. velezensis* YNK-FB0059 genome with four other classic *Bacillus* strains showed that the YNK-FB0059 genome is larger than that of strain FZB42, although their G + C contents are similar. The average coding gene length in *B. velezensis* YNK-FB0059 is 931.75 bp. The complete genome sequence of *Bacillus velezensis* YNK-FB0059 has been deposited in the GenBank database under the accession number CP140613.1. Four prophages were predicted within the chromosome sequence. Additionally, five genomic islands were identified.

**Table 3:** Genomic features of the *B. velezensis* YNK-FB0059 and related members of the *Bacillus* genus.

Features	*B. velezensis*			*B. amyloliquefaciens* DSM7	*B. subtilis*168
	YNK-FB0059	SQR9	FZB42		
Genome size (bp)	4,043,200	4,117,023	3,918,589	3,980,199	4,214,630
G + C content (%)	46.39%	46.1%	46.4%	46.1%	43.5%
Protein-coding sequences	3,848	4,078	3,693	3,921	4,106
Average CDS size (bp)	931.75	916	933	888	895
Percent of coding region	88.83%	90.7%	88%	87%	87%
Plasmid	1	0	0	0	0
Number of tRNAs	86	72	89	94	86
Ribosomal RNA operons	9	7	10	10	10
Phage-associated genes	7	218	44	185	268
GenBank sequence	CP140613.1	CP006890	CP000560	FN597644.1	AL009126.3

#### 3.2.2 Functional annotation based on public databases (NR, COG, KEGG, and GO)

**Figure 4:**
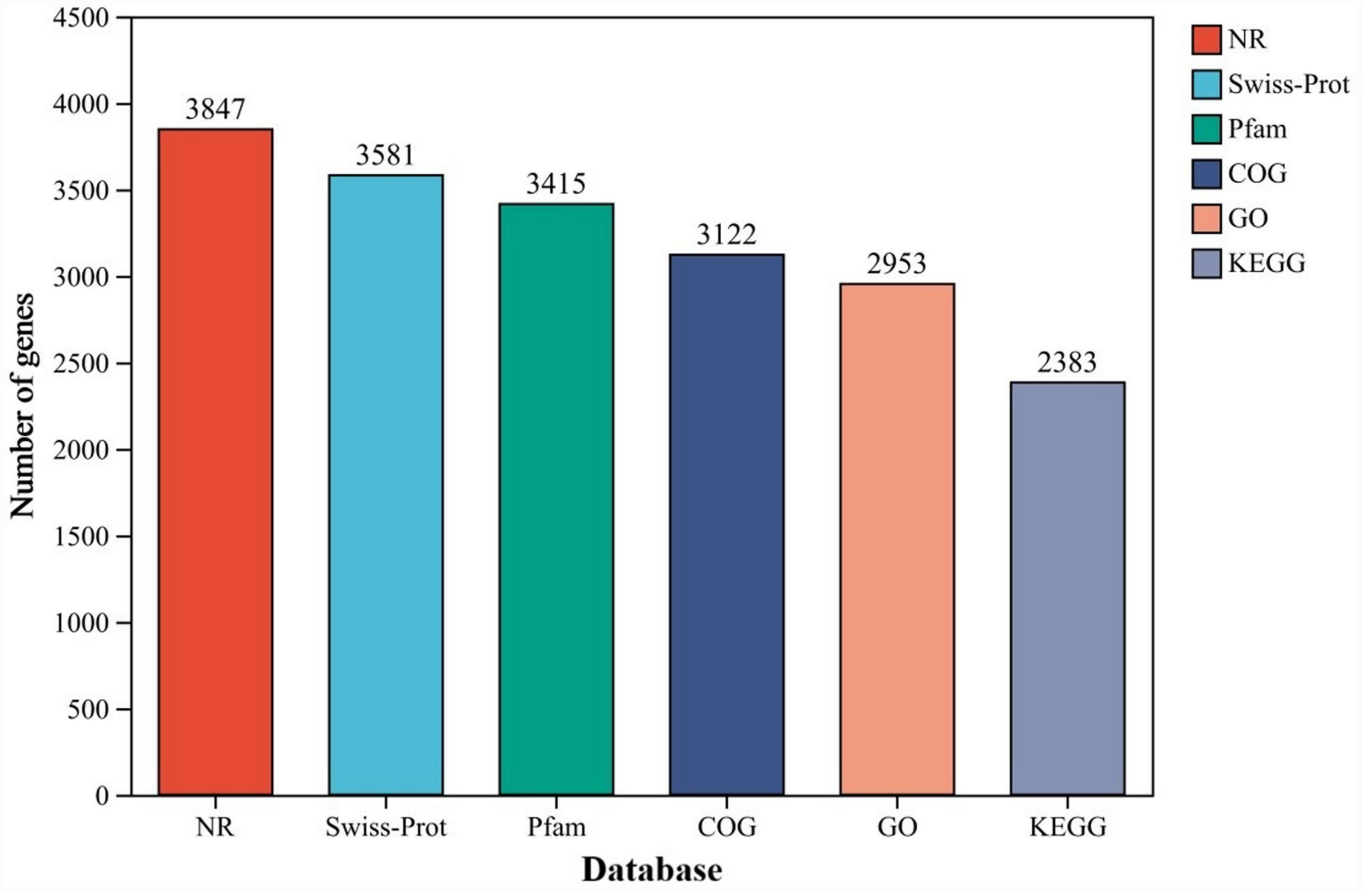
Distribution of gene function annotations of *B. velezensis* YNK-FB0059 across different databases. X-axis (Database): Database types used for functional annotation, including NR, Swiss-Prot, Pfam, COG, GO, and KEGG. Y-axis (Number of genes): Number of genes annotated in each database.

The predicted gene-protein sequences of *B. velezensis* YNK-FB0059 were compared against various functional databases, including NR, Swiss-Prot, Pfam, COG, GO, and KEGG, using a threshold E-value of ≤1e-5. The best match (with the highest score) for each sequence was selected for annotation, applying default thresholds of ≥40% identity and ≥40% coverage. The statistics of the final annotations are shown in Figure 4. The number of genes functionally annotated in the NR, Swiss-Prot, Pfam, COG, GO, and KEGG databases were 3,847, 3,581, 3,415, 3,122, 2,953, and 2,383, respectively. These correspond to 99.97, 93.06, 88.75, 81.13, 76.74, and 61.93% of the total predicted genes.

**Figure 5:**
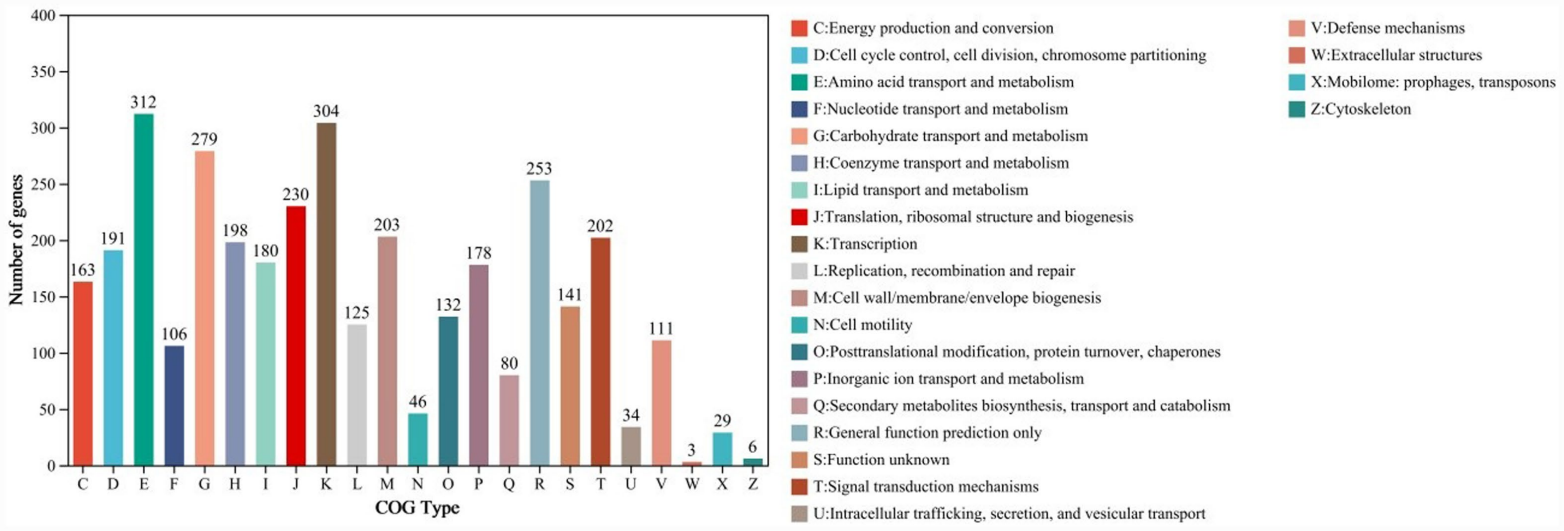
Functional annotation results of the *B. velezensis* YNK-FB0059 genome based on the COG database. X-axis (COG type): Categories of COG functional classification, such as amino acid transport and metabolism, transcription, carbohydrate transport and metabolism, etc. Y-axis (Number of genes): Number of genes annotated in each functional category.

Proteins encoded in the *B. velezensis* YNK-FB0059 genome with biological functions were annotated against the COG database, resulting in the annotation of 3,122 protein-coding genes (Figure 5). The functional annotation results were categorized into 23 classes, containing 111, 29, 34, 132, 46, 6, 191, 3, 203, 202, 304, 230, 125, 80, 198, 106, 178, 163, 279, 253, and 141 genes, respectively. Among these, “Amino acid transport and metabolism” was the most abundant category, with 312 genes, accounting for 9.99% of the total annotated genes. This was followed by “Transcription” with 304 genes (9.74%), “Carbohydrate transport and metabolism” with 279 genes (8.94%), “General function prediction only” with 253 genes (8.11%), and “Translation, ribosomal structure and biogenesis” with 230 genes (7.37%).

**Figure 6:**
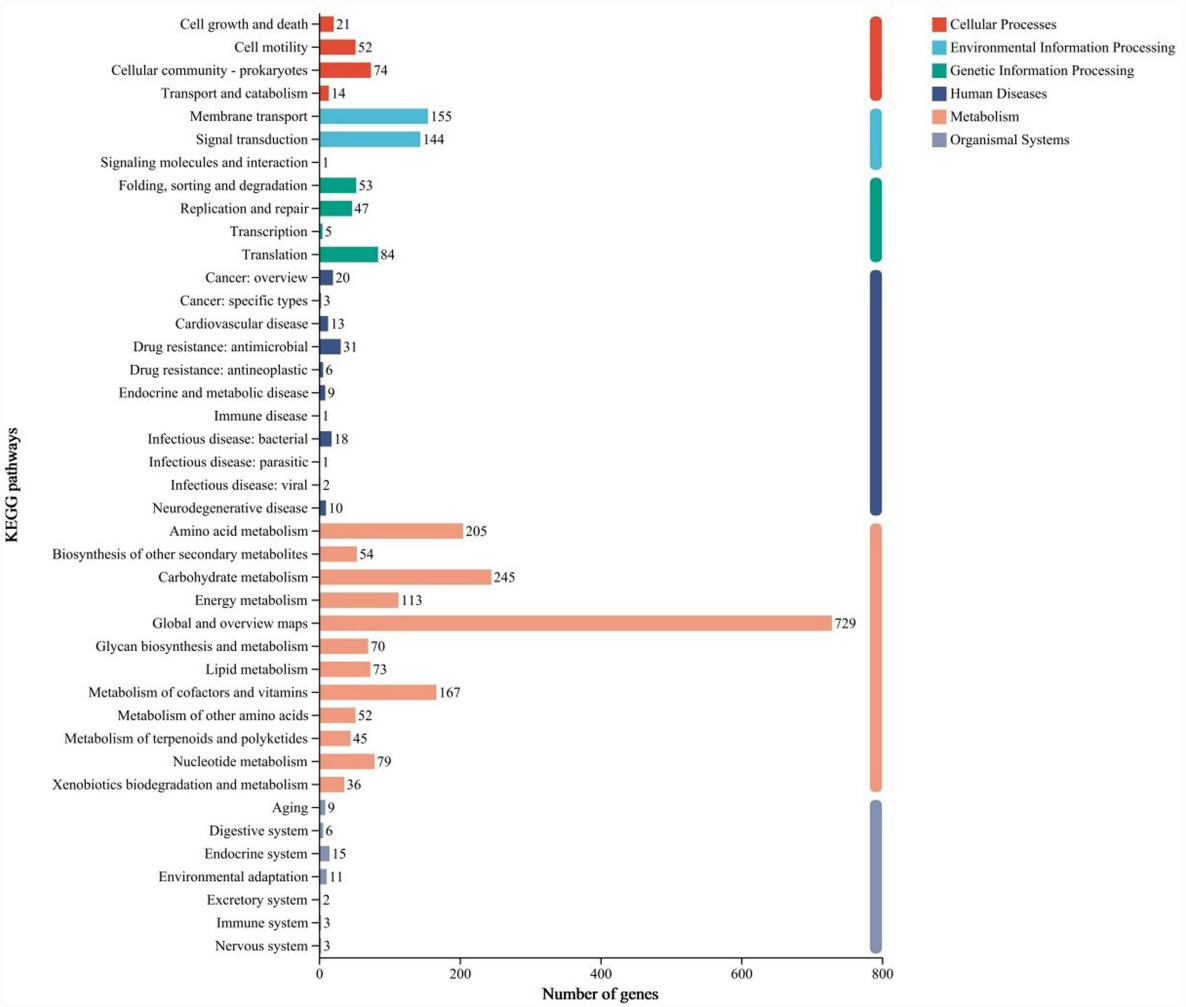
Functional annotation results of the *B. velezensis* YNK-FB0059 genome based on the KEGG database. Y-axis (KEGG pathways): Categories of metabolic or functional pathways to which the genes are assigned. X-axis (number of genes): Number of genes annotated in each pathway category.

A total of 2,383 genes from the *B. velezensis* YNK-FB0059 genome were annotated in the KEGG database, categorized into 6 major classes and 41 subclasses (Figure 6). Among these, 725 genes were associated with pathways related to plant growth promotion and immunity enhancement, including Amino acid metabolism, Biosynthesis of other secondary metabolites, Energy metabolism, Metabolism of terpenoids and polyketides, Metabolism of other amino acids, Environmental adaptation, and Carbohydrate metabolism.

**Figure 7:**
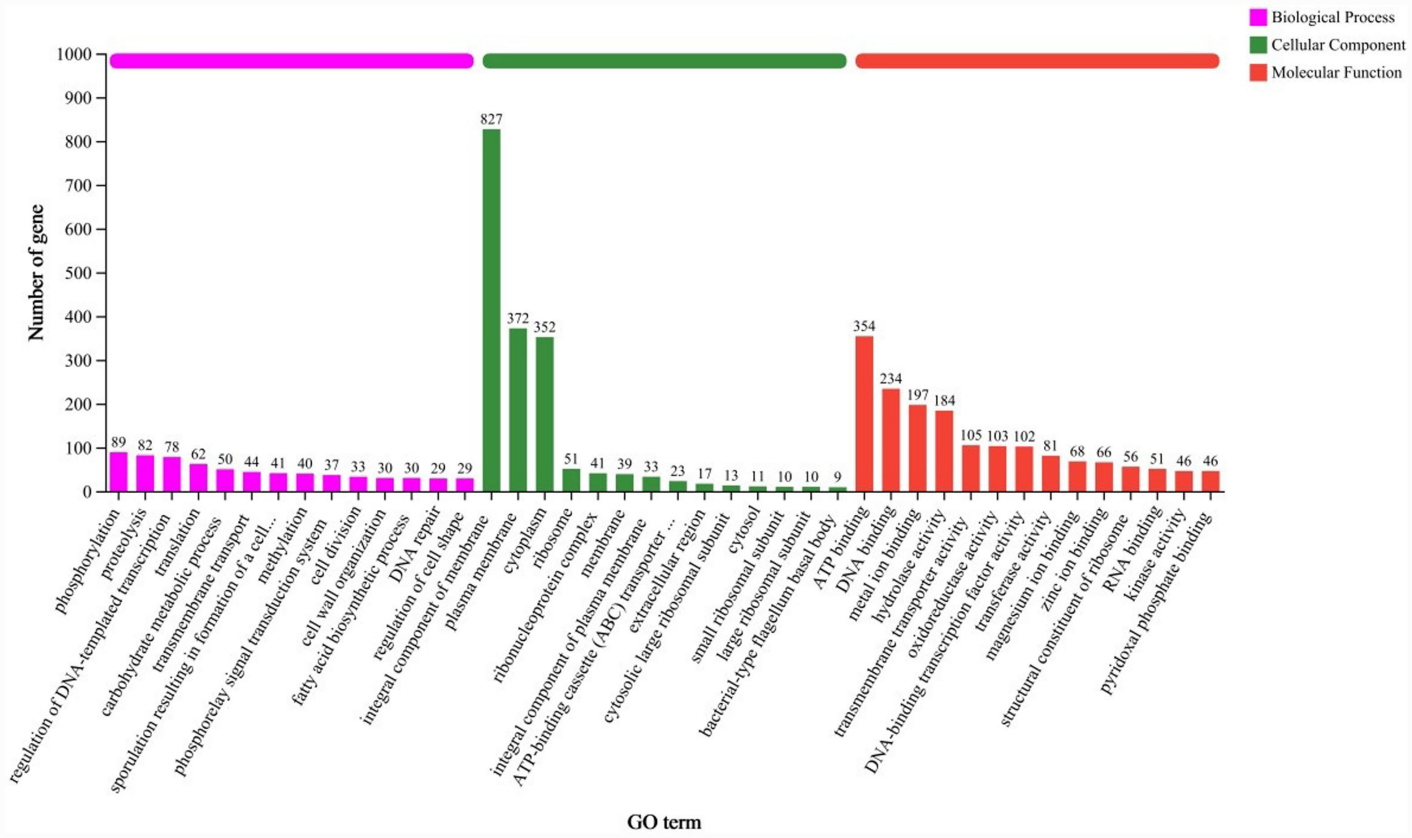
Functional annotation results of the GO database for the *B. velezensis* YNK-FB0059 genome. Y-axis (Number of genes): Number of genes annotated in each GO functional category. X-axis (GO Categories): GO functional categories, including biological process, cellular component, and molecular function.

Amino acid sequences of *B. velezensis* YNK-FB0059 were compared and statistically analyzed against the GO database to determine the distribution of functional genes in this strain. A total of 2,953 genes were annotated in the GO database. The GO database annotates proteins based on three aspects: Cellular Component, Biological Process, and Molecular Function (Figure 7). The Biological Process, Cellular Component, and Molecular Function branches were further divided into 14 subcategories each, totaling 42 subcategories. A total of 1,936 gene annotations were associated with the Cellular Component category. The most abundant subcategories were “integral component of membrane” (827 genes), “plasma membrane” (372 genes), and “cytoplasm” (352 genes). For the Biological Process category, 2,281 genes were annotated. The most represented processes were “phosphorylation” and “proteolysis,” with a combined total of 171 genes annotated to these specific terms. Regarding Molecular Function, there were 4,641 annotation instances (note: a single gene can have multiple molecular function annotations). The most prevalent functions were “ATP binding” (354 genes), “DNA binding” (234 genes), and “metal ion binding” (197 genes).

#### 3.2.3 CAZyme annotation

**Figure 8:**
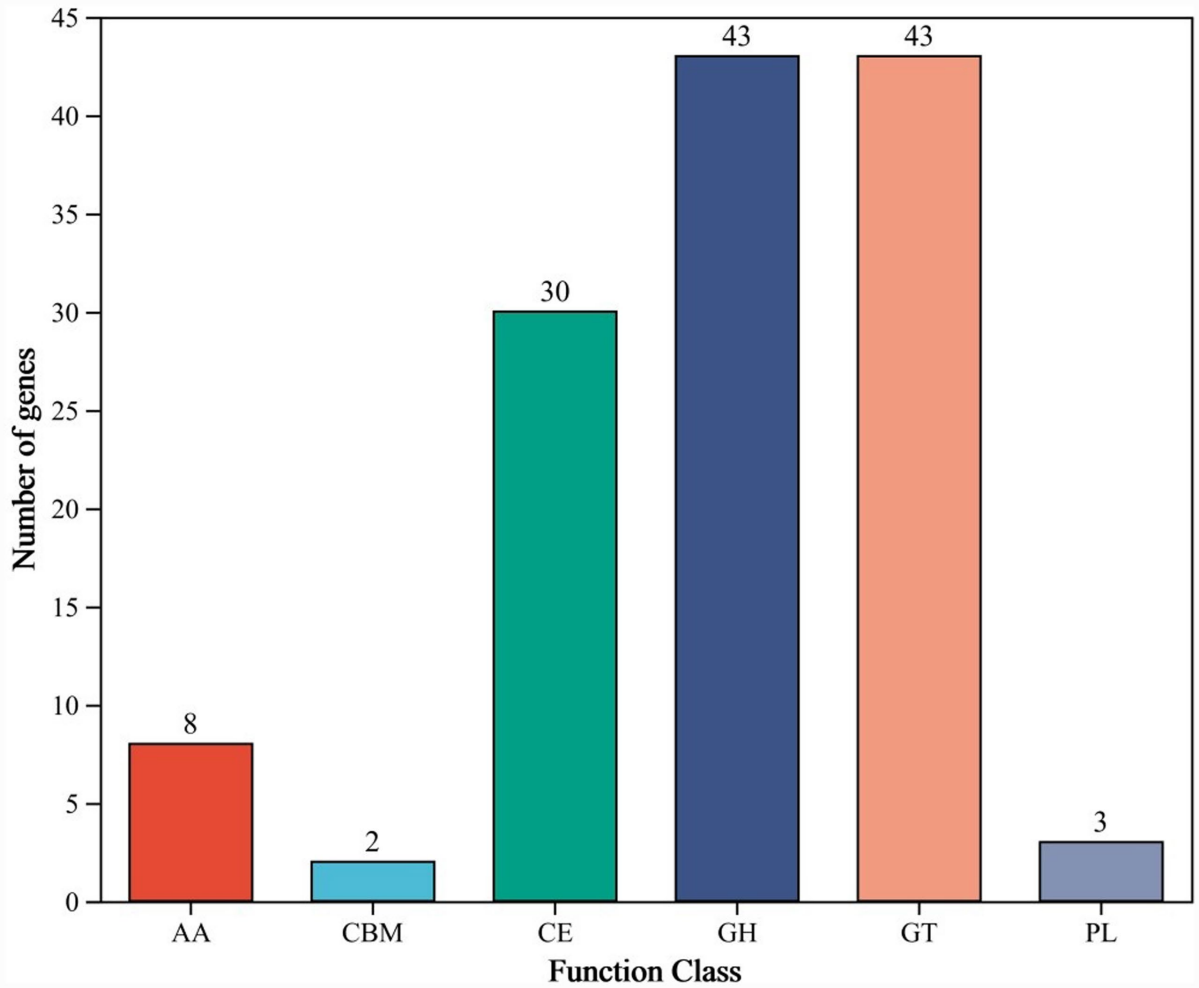
Functional annotation results of the CAZy database for the *B. velezensis* YNK-FB0059 genome. Y-axis (number of genes): Number of genes annotated in each CAZy functional category. X-axis (function class): CAZy functional categories, including AA (auxiliary activities), CBM (carbohydrate-binding modules), CE (carbohydrate esterases), GH (glycoside hydrolases), GT (glycosyl transferases), and PL (polysaccharide lyases).

Comparison of the genomic sequence against the CAZy database revealed 129 genes encoding protein domains belonging to CAZy families in the *B. velezensis* YNK-FB0059 genome (Figure 8). These include 43 Glycoside Hydrolases (GHs), 43 Glycosyl Transferases (GTs), 30 Carbohydrate Esterases (CEs), 8 Auxiliary Activities (AAs), 3 Polysaccharide Lyases (PLs), and 2 Carbohydrate-Binding Modules (CBMs). Furthermore, the genome of *B. velezensis* YNK-FB0059 was found to harbor multiple genes encoding enzymes such as endo-1,4-*β*-glucanase (endo-1,4-β-D-glucanase), β-glucosidase (a type of β-glycosidase), and *α*-amylase (encoded by amyE). These enzymes are involved in the decomposition and utilization of plant disease residues—comprising sugars and proteins—present in the soil.

### 3.3 Predictive analysis of plant growth–promoting traits

Based on genome annotation and comparative analyses, the potential plant growth–promoting traits of strain YNK-FB0059 were systematically predicted, including secondary metabolite biosynthesis, nutrient acquisition, phytohormone-related pathways, and plant immunity modulation.

#### 3.3.1 Secondary metabolite biosynthetic potential related to biocontrol

Comparative analysis using the KEGG database revealed that the genome of strain YNK-FB0059 possesses complete biosynthetic pathways for antibiotics and their precursors, including Bacilysin, Siderophore, Surfactin, Lichenysin D, Mycosubtilin, Iturin A, Bacillomycin D, Fengycin, Streptomycin, and the Terpenoid backbone biosynthesis pathway (Supplementary Figures S1–S5). Furthermore, analysis based on the antiSMASH database identified 15 biosynthetic gene clusters (BGCs) in the YNK-FB0059 genome (Table 4; Supplementary Figure S6). These include 4 Non-Ribosomal Peptide Synthetase (NRPS) clusters, 1 phosphonate cluster, 1 PKS-like cluster, 2 terpene clusters, 4 trans-AT PKS clusters, 2 Type III PKS (T3PKS) clusters, 1 betalactone cluster, 2 terpene-precursor clusters, 1 class II lanthipeptide cluster, and 1 cluster of other potential secondary metabolite type. Among these, 8 BGCs showed high similarity to known clusters responsible for the synthesis of surfactin, macrolactin H, bacillaene, fengycin, difficidin, bacillibactin, bacilysin, and mersacidin. This indicates that strain YNK-FB0059 has the genetic capacity to produce a diverse array of antimicrobial compounds. The MIBiG database was further utilized to predict the types of compounds with the highest synthesis similarity, as summarized in Table 5. Additionally, analysis using the BAGEL database revealed 5 RiPP (Ribosomally synthesized and post-translationally modified peptide) clusters in the *B. velezensis* YNK-FB0059 genome, predicted to form LCI, Colicin, Mersacidin, Amylocyclicin, and ComX3 (Table 6).

**Table 4:** Predicted secondary metabolite biosynthetic gene clusters (BGCs) in *Bacillus velezensis* YNK-FB0059 based on antiSMASH analysis.

Cluster ID	From	To	Cluster type	Similarity confidence	Most similar known cluster	
1	314,392	379,799	NRPS	High	Surfactin	NRPS: Type I
2	681,973	694,114	phosphonate	–	–	–
3	983,577	1,024,821	PKS-like	–	–	–
4	1,107,137	1,127,877	terpene	–	–	–
5	1,414,644	1,502,874	transAT-PKS	High	Macrolactin H	PKS
6	1,723,893	1,833,992	transAT-PKS, T3PKS, NRPS	High	Bacillaene	NRPS: Type I + PKS: Type I
7	1,900,836	2,038,666	NRPS, transAT-PKS, betalactone	High	Fengycin	NRPS: Type I
8	2,064,493	2,086,376	terpene	–	–	–
9	2,136,753	2,177,853	T3PKS	–	–	–
10	2,337,069	2,443,242	transAT-PKS	High	difficidin	PKS
11	2,466,570	2,487,460	terpene-precursor	–	–	–
12	3,066,178	3,131,535	terpene-precursor, NRP-metallophore, NRPS, RiPP-like	High	bacillibactin	NRPS: Type I
13	3,412,647	3,484,145	NRPS	–	–	–
14	3,686,371	3,727,789	other	High	bacilysin	Other: other
15	3,882,847	3,906,035	lanthipeptide-class-ii	High	mersacidin	Ribosomal: RiPP: Lanthipeptide

**Table 5:** Annotation of secondary metabolite biosynthetic gene clusters in *B. velezensis* YNK-FB0059 based on the MIBiG database.

Reference	Type	Similarity score	Compound(s)
BGC0000433.5	NRPS	0.83	Surfactin
BGC0000897.5	Other	0.47	Dehydrophos
BGC0002276.2	NRPS	0.55	Choline
BGC0002458.3	NRPS	0.49	Alazopeptin
BGC0000181.5	PKS	0.9	Macrolactin H
BGC0001089.5	NRPS, PKS	1.66	Bacillaene
BGC0001090.5	NRPS, PKS	1.68	Bacillomycin D
BGC0002173.2	saccharide, terpene	0.46	Fumihopaside A, compound 3, 21-β-H-hopane-3beta, 22-diol
BGC0000282.3	PKS	0.55	2-methoxy-5-methyl-6-(13-methyltetradecyl)-1,4-benzoquinone, 2-methoxy-5-methyl-6-(13-methyltetradecyl)phenol
BGC0000176.5	PKS	0.92	Difficidin
BGC0002283.2	terpene	0.49	Sodorifen
BGC0000616.4	ribosomal	2.45	Amylocyclicin
BGC0002135.2	NRPS	0.74	Bovienimide A
BGC0001184.4	other	0.97	Bacilysin
BGC0000527.5	ribosomal	1.00	Mersacidin

**Table 6:** Predicted ribosomally synthesized and post-translationally modified peptide (RiPP) gene clusters in *B. velezensis* YNK-FB0059 based on BAGEL analysis.

AOI ID	Start	End	Class
Chromosome.0. AOI_01	292,671	312,806	132.2; LCI
Chromosome.0. AOI_02	734,340	754,754	11.3; Colicin
Chromosome.0. AOI_03	3,881,060	3,904,972	62.1; Mersacidin
Chromosome.0. AOI_04	3,116,199	3,136,388	266.1; Amylocyclicin
Chromosome.0. AOI_05	3,066,015	3,086,177	320.1; ComX3

Furthermore, we employed PRISM to predict the structures of genetically encoded natural products in the *B. velezensis* YNK-FB0059 genome. The results identified 13 predicted compound clusters, including 4 Nonribosomal Peptides (NRPs), 4 Polyketides (PKs), 1 Class II/III Bacteriocin, 1 ComX pheromone, 1 Bacilysin, 1 head-to-tail cyclized bacteriocin, and 1 Class II Lanthipeptide (Table 7; Supplementary Figure S7).

**Table 7:** Predicted types of secondary metabolites encoded in the genome of *B. velezensis* YNK-FB0059 based on PRISM analysis.

Clusters	Cluster type
Cluster 1	Class II/III Confident Bacteriocin
Cluster 2	nonribosomal peptide
Cluster 3	polyketide
Cluster 4	polyketide、nonribosomal peptide
Cluster 5	polyketide、nonribosomal peptide
Cluster 6	nonribosomal peptide
Cluster 7	polyketide
Cluster 8	ComX
Cluster 9	nonribosomal peptide
Cluster 10	bacterial head-to-tail cyclized peptide
Cluster 11	nonribosomal peptide
Cluster 12	Bacilysin
Cluster 13	class II lantipeptide

#### 3.3.2 Prediction of genes involved in nutrient acquisition, plant growth promotion, and immunity enhancement

Genomic analysis of strain YNK-FB0059 identified two major categories of gene clusters closely associated with plant interactions, revealing the molecular basis for its dual potential in growth promotion and biocontrol.

On one hand, the strain possesses a comprehensive capacity for plant immunity activation (Supplementary Table S1). Its genome encodes various Microbe-Associated Molecular Patterns (MAMPs), including flagellin (FliC), bacterial chemotaxis proteins (e.g., MotA/B, CheA/Y), and surfactin/lichenysin synthetases (LicA/B/C), which can be recognized by plants to trigger basal immunity. Concurrently, the strain produces cell wall-degrading enzymes, such as pectate lyase (Pel) and endoglucanase, which release oligosaccharide fragments from plant or pathogen cell walls acting as elicitors. Furthermore, two-component systems (e.g., PhoPR, BceSR) and cell membrane modification genes (e.g., MprF, DltA-D) equip the strain with the ability to sense environmental stresses and evade host immune clearance, underscoring its strong environmental adaptability.

On the other hand, the strain also harbors multiple direct pathways for promoting plant growth (Supplementary Table S2). These include efficient nutrient transport systems for zinc, phosphate, oligopeptides, various amino acids, and sugars, which enhance rhizosphere nutrient cycling. Complete nitrogen metabolism pathways (e.g., nitrate reductase NasA, glutamine synthetase GlnA) and sulfur metabolism pathways (e.g., cysteine synthase CysK) help increase the levels of plant-available nutrients in the soil. Its complete tryptophan biosynthesis pathway (TrpA-F) provides precursors for the plant hormone auxin (IAA), indirectly stimulating root development. Additionally, the presence of multiple catalase (KatE) and cytochrome bd oxidase (CydAB) genes confers robust antioxidant and stress tolerance capabilities, facilitating stable colonization in the rhizosphere.

In summary, these genes collectively constitute the intrinsic mechanism by which strain YNK-FB0059 synergistically promotes plant health through activating systemic resistance, enhancing nutrient acquisition, and improving stress tolerance. This provides a solid genetic foundation for its development into an efficient and multifunctional microbial inoculant.

### 3.4 Screening of plant growth–promoting activity

To validate the plant growth–promoting potential predicted based on genome analysis, a series of *in vitro* and *in vivo* functional assays were conducted to evaluate nutrient transformation ability, phytohormone production, biofilm formation, antagonistic activity, and plant growth promotion.

#### 3.4.1 Antifungal activity and inhibition spectrum

**Figure 9:**
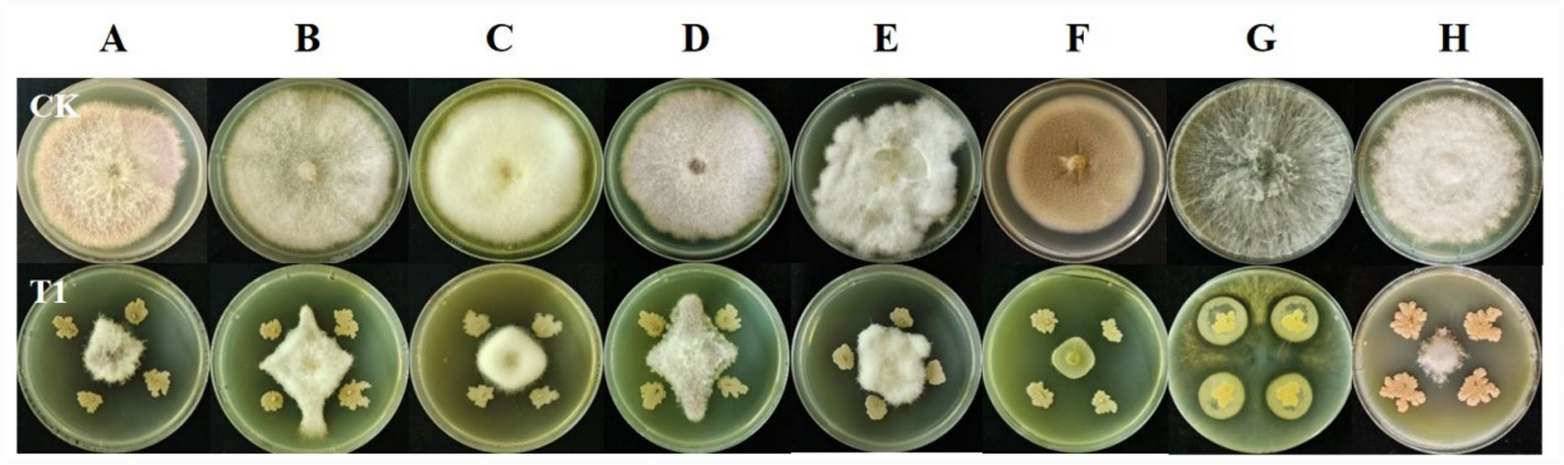
Broad-spectrum determination of antimicrobial activity of *B. velezensis* YNK-FB0059. **(A)** *Fusarium acuminatum*; **(B)** *Fusarium oxysporum* f.sp. *cubense*; **(C)** *Fusarium solani*; **(D)** *Fusarium graminearum*; **(E)** *Fusarium equiseti*; **(F)** *Phoma matteuciicola*; **(G)** *Diaporthe eres*; **(H)** *Phytophthora nicotianae*; CK: control group inoculated only with the pathogen; T1: plate confrontation treatment with strain YNK-FB0059.

The antifungal activity of strain YNK-FB0059 against various indicator pathogens was determined using the plate confrontation assay. As shown in Figure 9 and Table 8, strain YNK-FB0059 exhibited the highest inhibitory activity against the Phoma leaf spot pathogen of *Amomum tsaoko*, with an inhibition rate of 88.6%.

**Table 8:** Inhibition rate of strain YNK-FB0059 against pathogenic fungi.

Pathogenic fungi	Inhibition ratio (%)
*Fusarium acuminatum*	85.3 ± 1.22b
*Fusarium oxysporum* f.sp.cubense	77.8 ± 0.56d
*Fusarium solani*	82.6 ± 0.98bc
*Fusarium graminearum*	83.5 ± 1.02b
*Fusarium equiseti*	80.1 ± 1.11c
*Phoma matteuciicola*	88.6 ± 0.66 a
*Diaporthe eres*	78.2 ± 0.87d
*Phytophthora nicotianae*	86.9 ± 0.23b

Data are mean ± standard error of three independent biological replicates. Different letters indicate significant differences among treatments based on one-way ANOVA (*p* < 0.05).

Furthermore, it demonstrated strong antifungal activity against other pathogenic fungi, including *Fusarium acuminatum*, *Fusarium oxysporum* f. sp. cubense (banana Fusarium wilt pathogen), *Fusarium solani*, *Fusarium graminearum*, *Fusarium equiseti*, *Diaporthe eres* (branch blight pathogen of *Amomum tsaoko*), and *Phytophthora nicotianae*. The corresponding inhibition rates were 85.3, 77.8, 82.6, 83.5, 80.1, 78.2, and 86.9%, respectively. These results indicate that strain YNK-FB0059 possesses broad-spectrum and potent antifungal activity. Statistical analysis showed that inhibition rates differed significantly among treatments (one-way ANOVA, *p* < 0.05).

#### 3.4.2 Effects on mycelial growth and conidial germination

**Figure 10:**
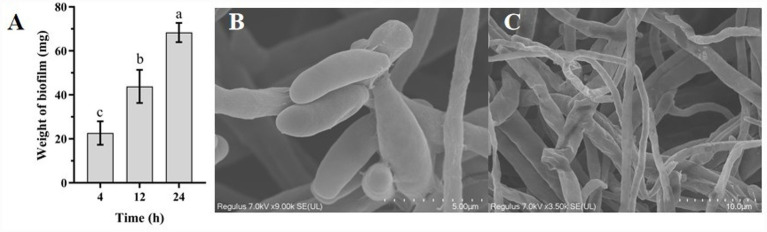
Effect of strain *YNK-FB0059* on spore germination and mycelial growth of *Phoma matteuciicola*. **(A)** Conidial germination rate; **(B)** conidial morphology; **(C)** mycelial morphology observed by SEM. Data represent mean ± SE of three independent replicates. Different letters indicate significant differences (one-way ANOVA, *p* < 0.05).

Evaluation of the impact of strain YNK-FB0059 fermentation broth on the conidial germination of the Phoma leaf spot pathogen of *Amomum tsaoko* showed no significant effect on conidial morphology (Figure 10B). However, the fermentation broth significantly affected the conidial germination rate of the pathogen at all observed growth stages (*p* < 0.05; Figure 10A). The highest inhibition rate (68.35%) was observed at 24 h, while the lowest (22.66%) occurred at 4 h, with an intermediate rate of 43.84% at 12 h. Observation via scanning electron microscopy (SEM) revealed that, under the influence of strain YNK-FB0059, the pathogenic hyphae became blunted, curved, thinned, reduced in quantity, shortened, and fragmented (Figure 10C). These results demonstrate that strain YNK-FB0059 can suppress pathogen germination and growth by inhibiting both conidial germination and the development of newly formed hyphae during the spore germination and mycelial growth stages.

#### 3.4.3 Nutrient transformation abilities

**Figure 11:**
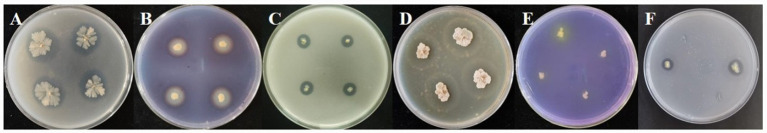
Qualitative assessment of plant growth–promoting traits of *B. velezensis* YNK-FB0059 using colorimetric plate assays. **(A)** Organic phosphorus solubilization; **(B)** siderophore secretion; **(C)** zinc solubilization; **(D)** potassium release; **(E)** sulfur oxidation; **(F)** nitrogen fixation.

Functional screening revealed that strain YNK-FB0059 not only exhibits broad-spectrum antimicrobial activity but also possesses the ability to solubilize organic phosphorus (Figure 11A), secrete siderophores (Figure 11B), solubilize zinc (Figure 11C), release potassium (Figure 11D), oxidize sulfur (Figure 11E), and fix nitrogen (Figure 11F). Furthermore, KEGG pathway analysis identified numerous genes in the YNK-FB0059 genome associated with nutrient transformation, including *nirD*, *nirB*, *nasA*, and *narK* involved in nitrogen fixation; *cysK*, *cysE*, *ssuB*, *ssuA*, *ssuC*, and *ssuD* involved in sulfur metabolism; and *menF*, *dhbF*, *entB*, *entE*, *entC*, and *entA* involved in siderophore biosynthesis. Complete pathways for nitrogen fixation and sulfur metabolism were also identified (Supplementary Figures S8, S9). The activities of organic phosphorus solubilization, potassium release, sulfur oxidation, nitrogen fixation, and zinc solubilization demonstrate the potential of YNK-FB0059 to function as a plant growth-promoting rhizobacterium (PGPR). This highlights its significant application potential in promoting plant growth, enhancing systemic resistance, and improving soil nutrient availability. Additionally, the secretion of siderophores enables the strain to suppress pathogenic microorganisms by competing for limited iron resources in the soil, thereby contributing to disease control.

#### 3.4.4 Biofilm formation and IAA production

**Figure 12:**
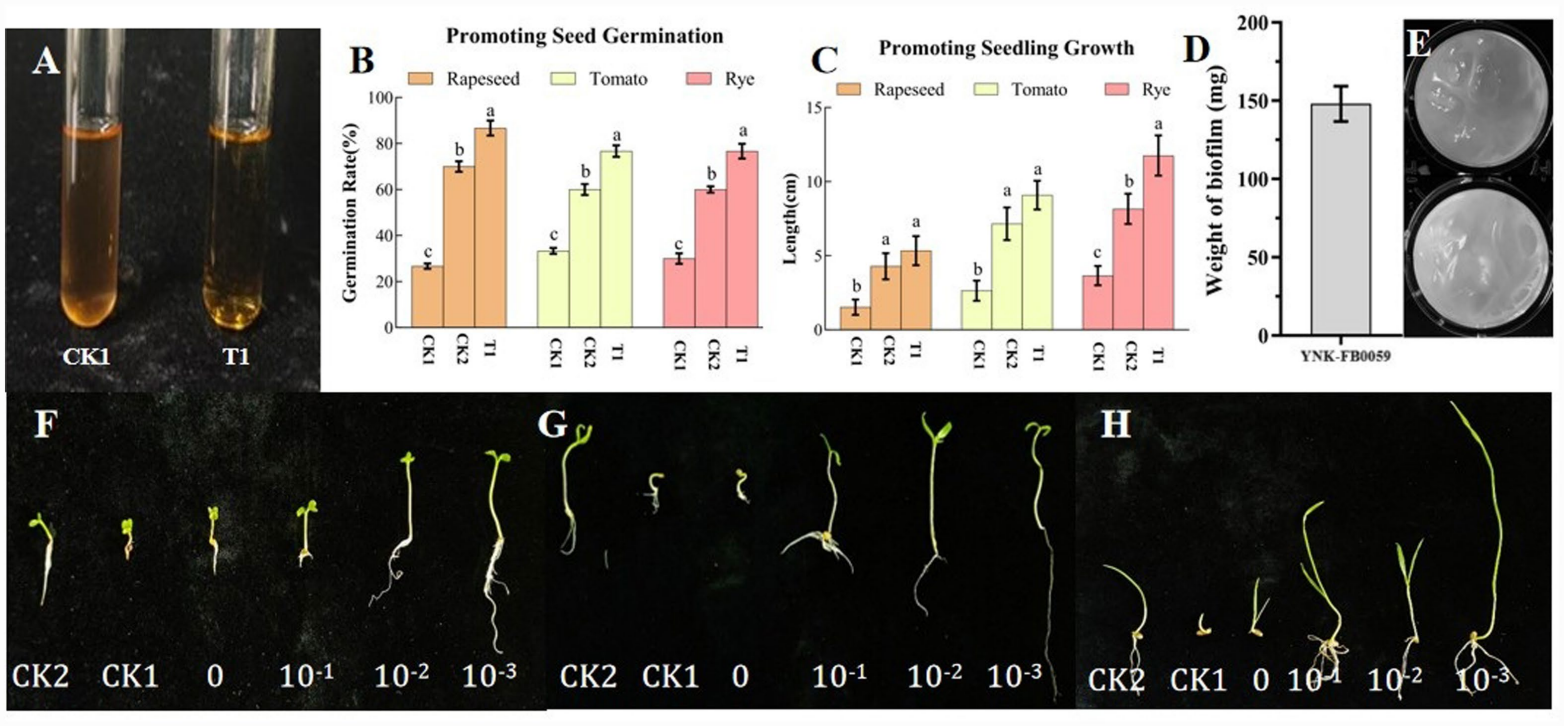
Biofilm formation, IAA production, and seed germination- and growth-promoting effects of strain YNK-FB0059. **(A)** IAA production (test tube); **(B)** Seed germination rate (rapeseed, tomato, rye; CK1: sterile water control, CK2: uninoculated medium control, T1: YNK-FB0059 treatment) **(C)** Seedling growth (rapeseed, tomato, rye; CK1: sterile water control, CK2: uninoculated medium control, T1: YNK-FB0059 treatment); **(D)** Biofilm biomass (bar graph); **(E)** Biofilm morphology in 6-well plate; **(F)** Rapeseed seed promotion; **(G)** Tomato seed promotion; **(H)** Rye seed promotion. Data represent mean ± SE of three independent biological replicates (*n* = 3). Different letters above bars indicate significant differences among treatments (one-way ANOVA, *p* < 0.05).

Biofilm formation is a key factor determining the colonization ability of functional strains on plant roots. The biofilm-forming capacity of *Bacillus velezensis* YNK-FB0059 was qualitatively and quantitatively analyzed using MSgg medium. After 16 h of incubation, YNK-FB0059 was observed to form extensive wrinkled colonies on MSgg medium (Figure 12E). For quantitative analysis, biofilms were collected from each well of a 6-well plate containing 10 mL of MSgg medium per well. The biomass of biofilm formed by YNK-FB0059 was 148 mg per well, representing the mean of three independent biological replicates (Figure 12D).

Qualitative experiments confirmed that strain YNK-FB0059 is capable of secreting indole-3-acetic acid (IAA) (Figure 12A). Using Sackowcki’s colorimetric method, the OD₅₃₅ value was measured and applied to the standard curve **y =** 0.0069x + 0.0058 (R^2^ = 0.9989), yielding an IAA secretion level of 42.55 μg·mL^−1^ at 48 h. KEGG database comparison and genomic annotation further revealed that the YNK-FB0059 genome harbors multiple genes involved in IAA biosynthesis, such as trpA, trpB, trpC, trpD, and trpE, along with a complete IAA synthesis pathway (Supplementary Figure S10).

In addition, indoor experiments were conducted to evaluate the growth-promoting effects of the strain on rapeseed, tomato, and rye seeds (Figures 12F,G,H). The results demonstrated that strain YNK-FB0059 significantly enhanced seed germination rates and agronomic traits compared to the blank control (CK). Specifically, it increased the germination rate of rapeseed to 86.7% and whole plant length to 5.34 cm; improved tomato seed germination to 76.7% and plant length to 9.09 cm; and elevated rye germination to 76.7% with a plant length of 11.76 cm (Figures 12B,C).

In summary, these findings indicate that strain YNK-FB0059 not only possesses the potential for effective root colonization but also promotes seed germination and plant growth, underscoring its promise as a beneficial plant root-associated microorganism.

#### 3.4.5 Effects of *Bacillus velezensis* YNK-FB0059 on tomato seedling growth in pot experiments

A pot experiment was conducted to evaluate the growth-promoting effects of strain YNK-FB0059 on tomato seedlings. The results (Table 9; Supplementary Figure S11) demonstrated that, compared to the sterile water control (CK1) and sterile fermentation broth, inoculation with the diluted fermentation broth of strain YNK-FB0059 significantly promoted the growth of tomato seedlings. This was manifested as increased plant height, enhanced primary root growth, higher fibrous root number, and greater above-ground fresh and dry weight.

**Table 9:** Effect of fermentation dilution of strain YNK-FB0059 on growth of tomato seedlings.

Treatment	Height (cm)	Stem thick (cm)	Root length (cm)	Root weight (g)	Fresh weight of above-ground part (g)	Dry weight of above-ground part (g)
0	50.13 ± 3.22ab	3.18 ± 0.02b	13.33 ± 2.63a	2.35 ± 0.08b	25.13 ± 2.66ab	7.69 ± 1.11b
10^−1^	56.62 ± 2.98a	3.56 ± 0.11a	16.52 ± 1.85a	3.15 ± 0.24a	29.33 ± 3.21a	10.85 ± 1.04a
10^−2^	54.63 ± 1.65a	3.19 ± 0.21b	16.18 ± 1.49a	3.22 ± 0.16a	28.54 ± 1.85a	10.27 ± 1.58a
10^−3^	55.36 ± 1.89a	3.21 ± 0.09b	15.86 ± 2.87a	3.36 ± 0.31a	29.21 ± 1.69a	10.48 ± 1.87a
CK2	47.31 ± 2.31b	2.45 ± 0.22c	15.96 ± 1.84a	2.11 ± 0.15b	23.59 ± 2.87b	7.11 ± 1.23b
CK1	47.22 ± 2.68b	2.66 ± 0.37c	12.05 ± 1.99a	1.94 ± 0.47b	22.36 ± 1.49b	6.98 ± 1.28b

Data are presented as mean ± SE. Different letters indicate significant differences between treatments (one-way ANOVA, *p* < 0.05).

These findings indicate that strain YNK-FB0059 has a notable growth-promoting effect on tomatoes and represents a valuable resource as an efficient plant growth-promoting strain.

## 4 Discussion

This study systematically characterized the genome of *B. velezensis* YNK-FB0059 and, through multi-dimensional functional validation, confirmed it as a multifunctional plant growth–promoting rhizobacterium (PGPR) integrating efficient biocontrol activity, multiple plant growth–promoting (PGP) traits, and strong rhizosphere colonization capacity.

Compared with the well-studied model strain *B. velezensis* FZB42, YNK-FB0059 exhibited a broader and more potent antifungal spectrum, showing particularly high inhibitory activity against Phoma matteuciicola (88.6%) and strong suppression of oomycete pathogens such as Phytophthora nicotianae, extending beyond the antifungal range commonly reported for FZB42 (Qiao et al., 2014).

Genome analysis revealed that *B. velezensis* YNK-FB0059 harbors 15 secondary metabolite biosynthetic gene clusters (BGCs), including NRPS-derived lipopeptides (surfactin, fengycin, bacillomycin D), polyketides (macrolactin H, bacillaene, difficidin), RiPPs (mersacidin), bacilysin, and lichenysin, indicating its potential to produce diverse antimicrobial compounds (Wang et al., 2023; Chen et al., 2025). Based on *in vitro* antifungal assays against eight plant pathogens, specific BGCs may correspond to observed inhibition. Lipopeptide BGCs (surfactin and fengycin) are likely major contributors to the inhibition of *Fusarium graminearum*, *F. solani*, and *Phoma matteuciicola*, as these lipopeptides can disrupt fungal cell membranes and inhibit hyphal growth and spore germination (Ongena and Jacques, 2008). In addition, the bacillomycin D BGC may play an important role in suppressing *Fusarium oxysporum* f. sp. *cubense* and *Phytophthora nicotianae*, consistent with reports that iturin-family lipopeptides exhibit strong antifungal and anti-oomycete activities (Harwood et al., 2018). Polyketide BGCs (macrolactin H, bacillaene, difficidin) together with other secondary metabolites (mersacidin, bacilysin, lichenysin) may act synergistically to enhance broad-spectrum antagonism (Kenfaoui et al., 2024). Overall, the diversity of BGCs aligns with the broad antifungal spectrum observed in vitro, providing a plausible genomic basis for the biocontrol potential of YNK-FB0059.

Beyond biocontrol potential, YNK-FB0059 displayed multiple direct plant growth–promoting traits, including phosphate solubilization, siderophore production, nitrogen metabolism, and indole-3-acetic acid (IAA) secretion.

Notably, the strain produced a relatively high level of IAA (42.55 μg·mL^−1^ within 48 h), which is higher than that reported for several previously described *B. velezensis* strains (He et al., 2024), highlighting its strong potential for promoting plant growth.

In addition, YNK-FB0059 exhibited a strong biofilm-forming capacity (148 mg dry weight), providing a crucial basis for effective root colonization and long-term functional stability in the rhizosphere, surpassing the biofilm production of strain SQR9 (Wu et al., 2018). His is consistent with previous reports showing that biofilm formation is essential for *Bacillus velezensis* root colonization and persistence in plant environments (Xu et al., 2019).

Previous studies have shown that *Bacillus* spp. exert biocontrol activity through multiple mechanisms, including the production of antimicrobial compounds such as lipopeptides that directly inhibit phytopathogens and the induction of systemic resistance in host plants. Consistent with these mechanisms, the genome of strain *YNK-FB0059* encodes flagellin, chemotaxis-related proteins, and other microbe-associated molecular patterns (MAMPs) that may contribute to plant immune activation via MAMP-triggered immunity. *Peng et al.* reviewed these biocontrol mechanisms of *Bacillus* in plant disease management, including ISR and biofilm-mediated colonization of the rhizosphere, supporting the potential multifaceted roles of *YNK-FB0059* in plant protection (Peng et al., 2025).

In intensive cropping systems, soil-borne diseases caused by *Fusarium* spp. are increasingly aggravated by continuous monocropping and excessive chemical fertilizer and pesticide inputs, leading to serious yield losses and ecological risks (Gao et al., 2025).

The strong inhibitory activity of YNK-FB0059 against multiple *Fusarium* species highlights its promising application for disease suppression in economically important crops such as tomato and banana, which is consistent with previous reports on the effectiveness of lipopeptide-producing *Bacillus* strains against *Fusarium* wilt (Meng et al., 2025).

Meanwhile, YNK-FB0059 exhibits efficient nutrient transformation capabilities, including phosphate solubilization, nitrogen fixation, and siderophore production. By promoting nutrient cycling and enhancing beneficial microbial populations in the rhizosphere, it not only suppresses pathogens but also contributes to long-term soil health, improves nutrient use efficiency, and helps reduce chemical fertilizer inputs, meeting the urgent needs of sustainable agricultural development (Li et al., 2025).

Against the background of global efforts to promote green and sustainable agriculture, the exploitation of indigenous multifunctional strains such as YNK-FB0059 is of great strategic significance. Long-term overuse of chemical pesticides and fertilizers has been shown to disrupt soil microbial communities and impair ecosystem services (Liang et al., 2025; Zhou et al., 2025).

Probiotic strains originating from local ecosystems often exhibit superior environmental adaptability and rhizosphere competitiveness. This is consistent with the view that screening functional microorganisms from crop-specific rhizospheres or native habitats is a key strategy for developing effective microbial inoculants (Zhang et al., 2024).

Overall, the multifunctional traits of YNK-FB0059—including broad-spectrum biocontrol, high IAA production, strong biofilm formation, and efficient nutrient transformation—underscore its practical potential as a biofertilizer or biocontrol agent in sustainable crop production systems. By combining direct pathogen antagonism, plant immune induction, and nutrient facilitation, these traits provide a solid foundation for its development in sustainable agriculture.

## 5 Conclusion

*Bacillus velezensis* YNK-FB0059 was identified through polyphasic taxonomy as a multifunctional rhizosphere probiotic strain possessing excellent biocontrol and plant growth-promoting potential. Whole-genome analysis revealed 14 secondary metabolite biosynthetic gene clusters encoding broad-spectrum antimicrobial compounds, including surfactin, fengycin, and bacillomycin, along with complete genetic pathways for nitrogen fixation, phosphate solubilization, siderophore production, and IAA biosynthesis.

*In vitro* experiments demonstrated strong inhibitory activity against eight plant pathogens (inhibition rates: 77.8–88.6%), effective suppression of spore germination, and deformation of pathogenic hyphae. The strain also exhibited multiple functional traits, including phosphate and potassium solubilization, nitrogen fixation, IAA secretion (42.55 μg·mL^−1^), and robust biofilm formation (148 mg). In pot experiments, it significantly promoted tomato growth.

The study confirms that strain YNK-FB0059 operates through a synergistic mechanism integrating biocontrol, growth promotion, and colonization, highlighting its great potential as a high-efficacy microbial inoculant.

Building on these findings, future work could focus on: (1) developing synergistic composite microbial agents by combining YNK-FB0059 with functionally complementary microbes; (2) optimizing fermentation, carrier, and preservation technologies to enhance product stability and practicality; (3) conducting large-scale field evaluations across multiple crops and soil types to provide robust data supporting commercialization; and (4) in-depth exploration of its active metabolites, including the isolation and identification of novel antimicrobial compounds, to provide candidate molecules for new biopesticide development.

## Data Availability

The original contributions presented in the study are included in the article/Supplementary material, further inquiries can be directed to the corresponding author/s.
