# Supplementary material for: Whole genome analysis of Bacillus velezensis YNK-FB0059 and its multifunctional plant growth-promoting and biocontrol potential
Source: Front Microbiol. 2026 Jan 30;17:1748090. doi: 10.3389/fmicb.2026.1748090 (PMC12901434; doi:10.3389/fmicb.2026.1748090)
Supplement: Supplementary file 1 [file Data_Sheet_1.docx]

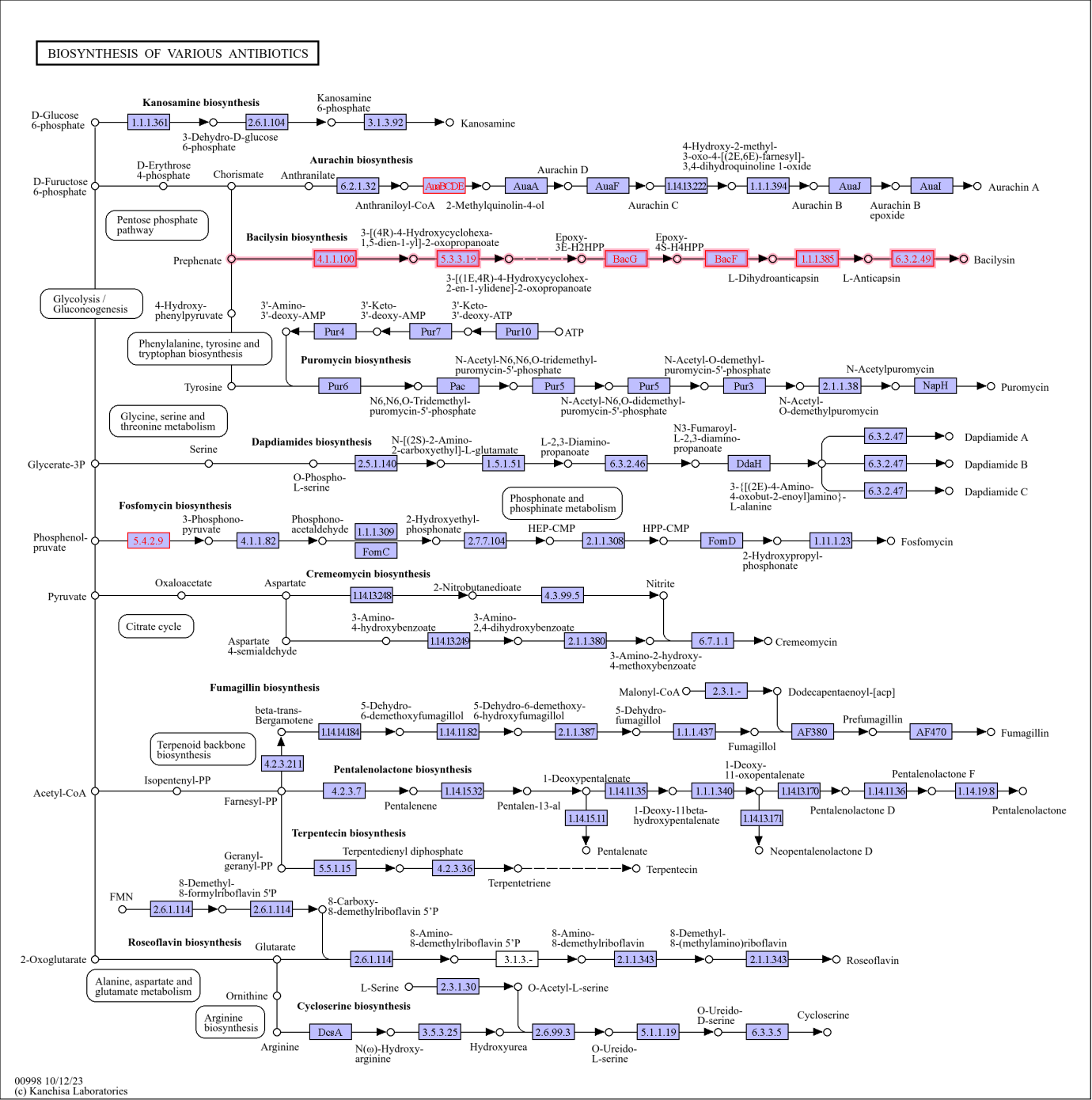


**Figure S1.** Genome circle map of *Bacillus velezensis YNK-FB0059*.


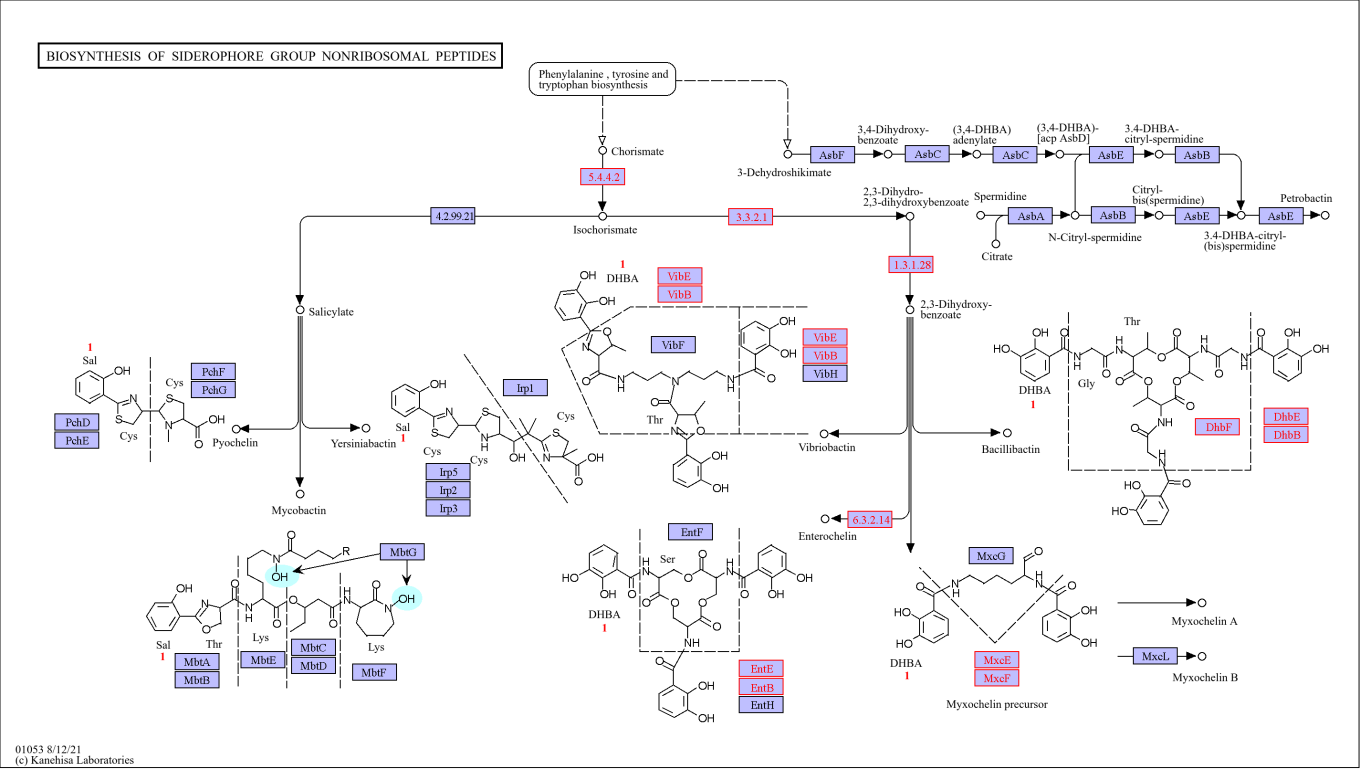


**Figure S2.** Biosynthesis of siderophore group nonribosomal peptides.


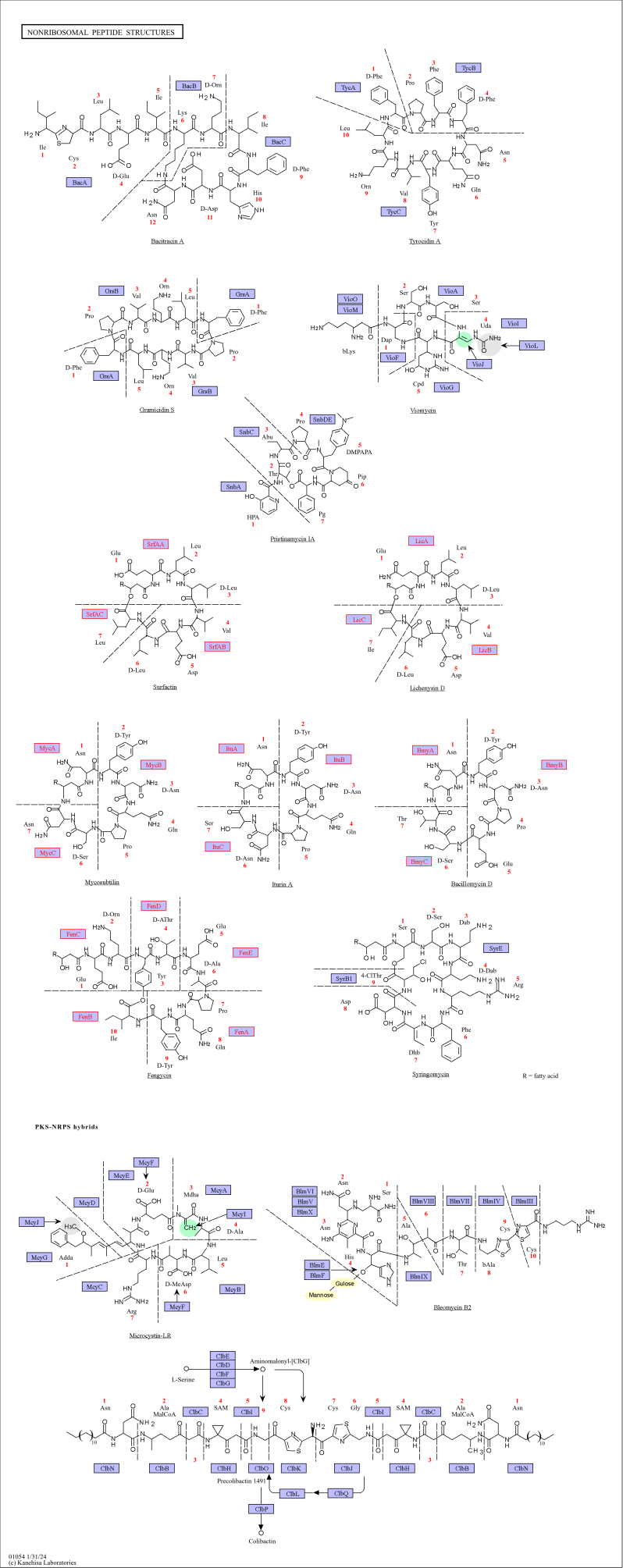


**Figure S3.** Nonribosomal peptide structures.


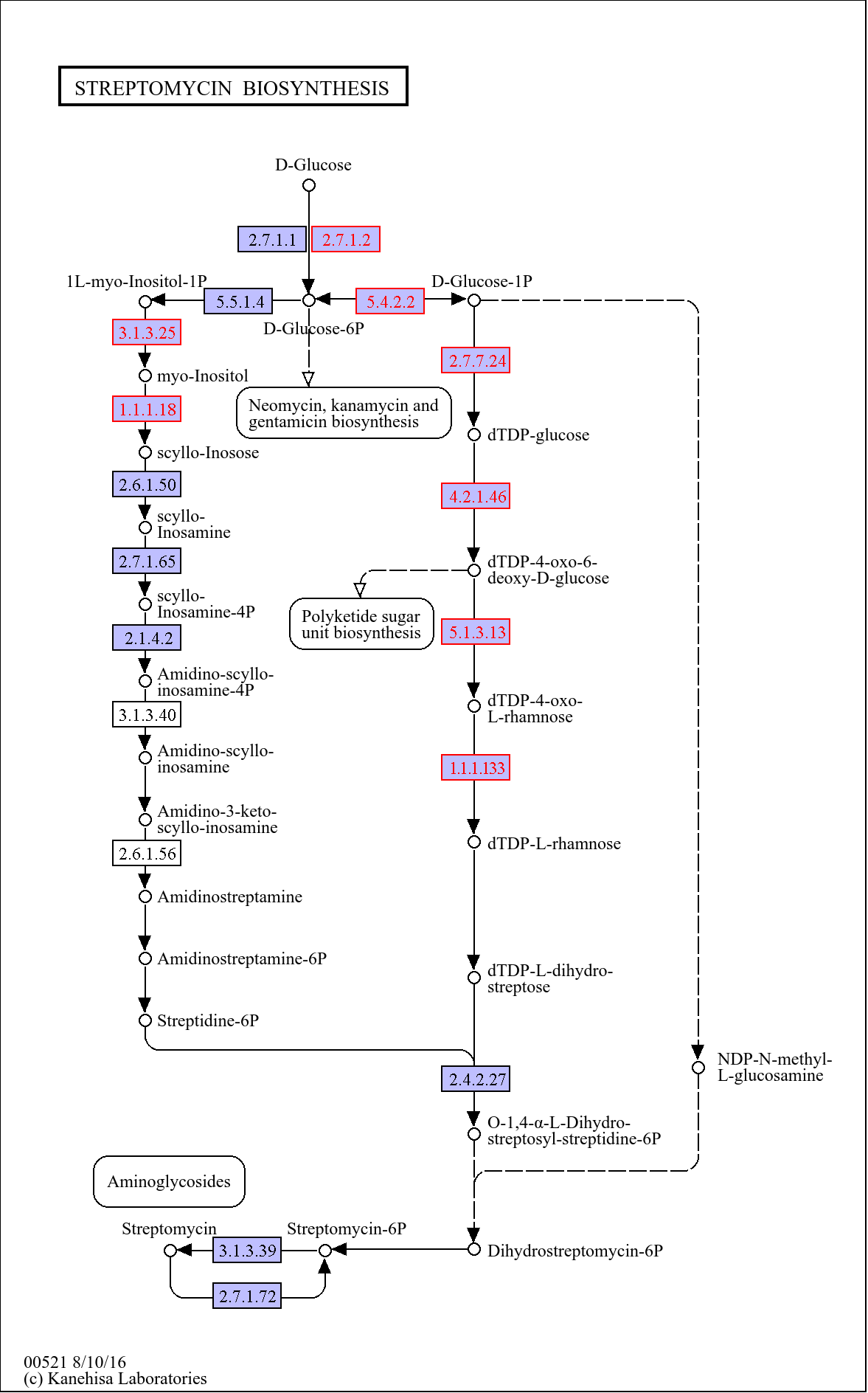


**Figure S4.** Streptomycin Biosynthesis.


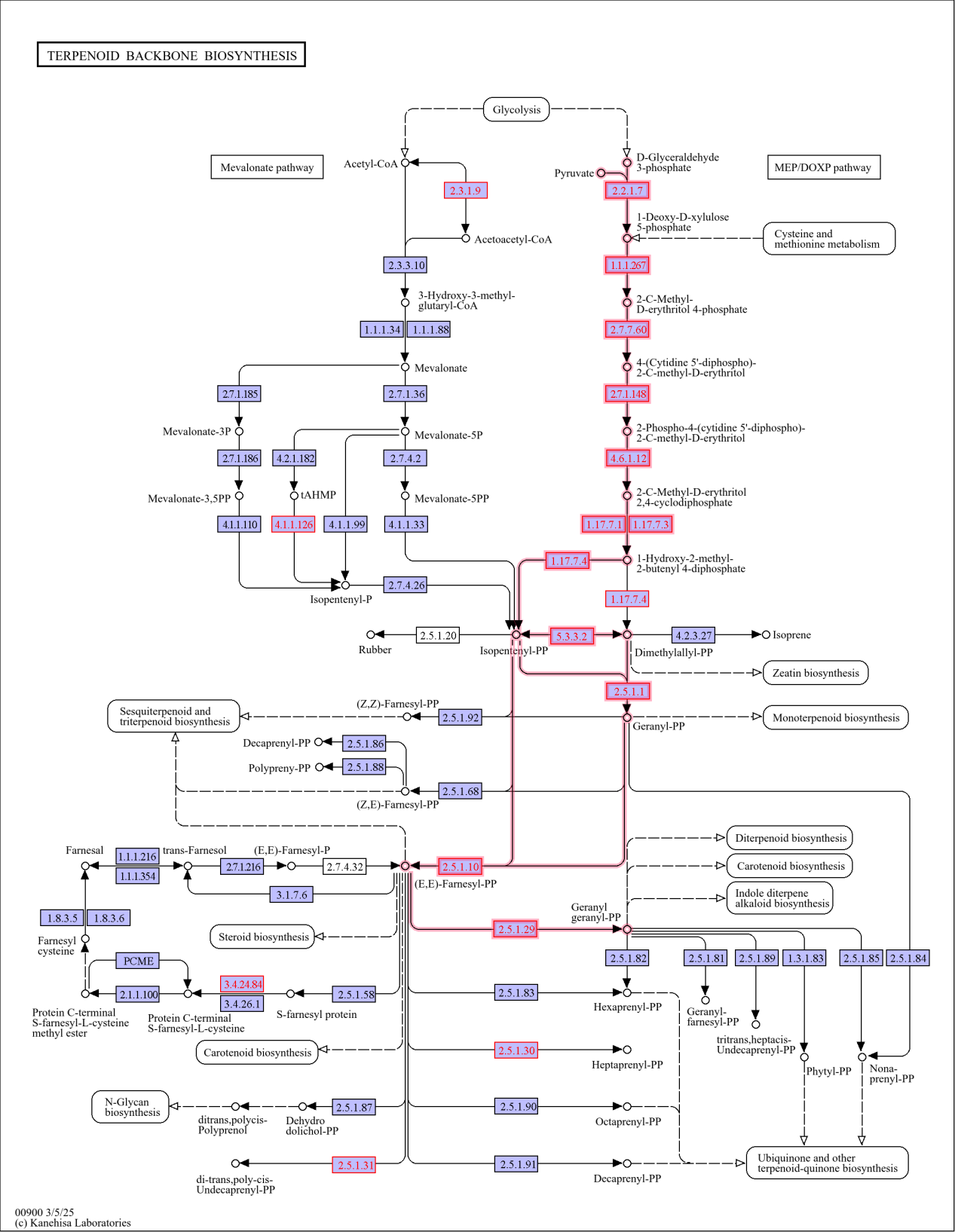


**Figure S5.** Terpenoid backbone biosynthesis.


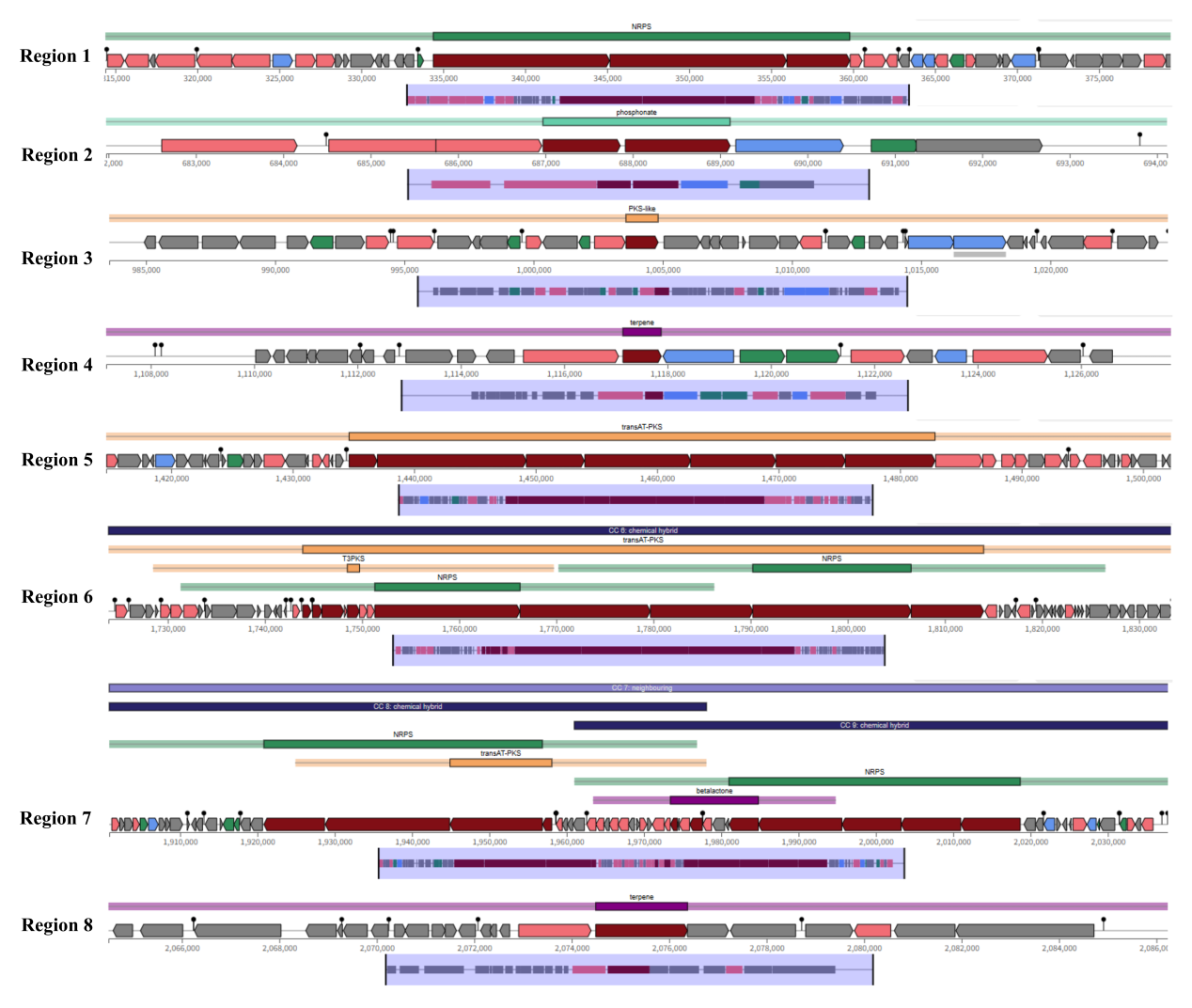


**
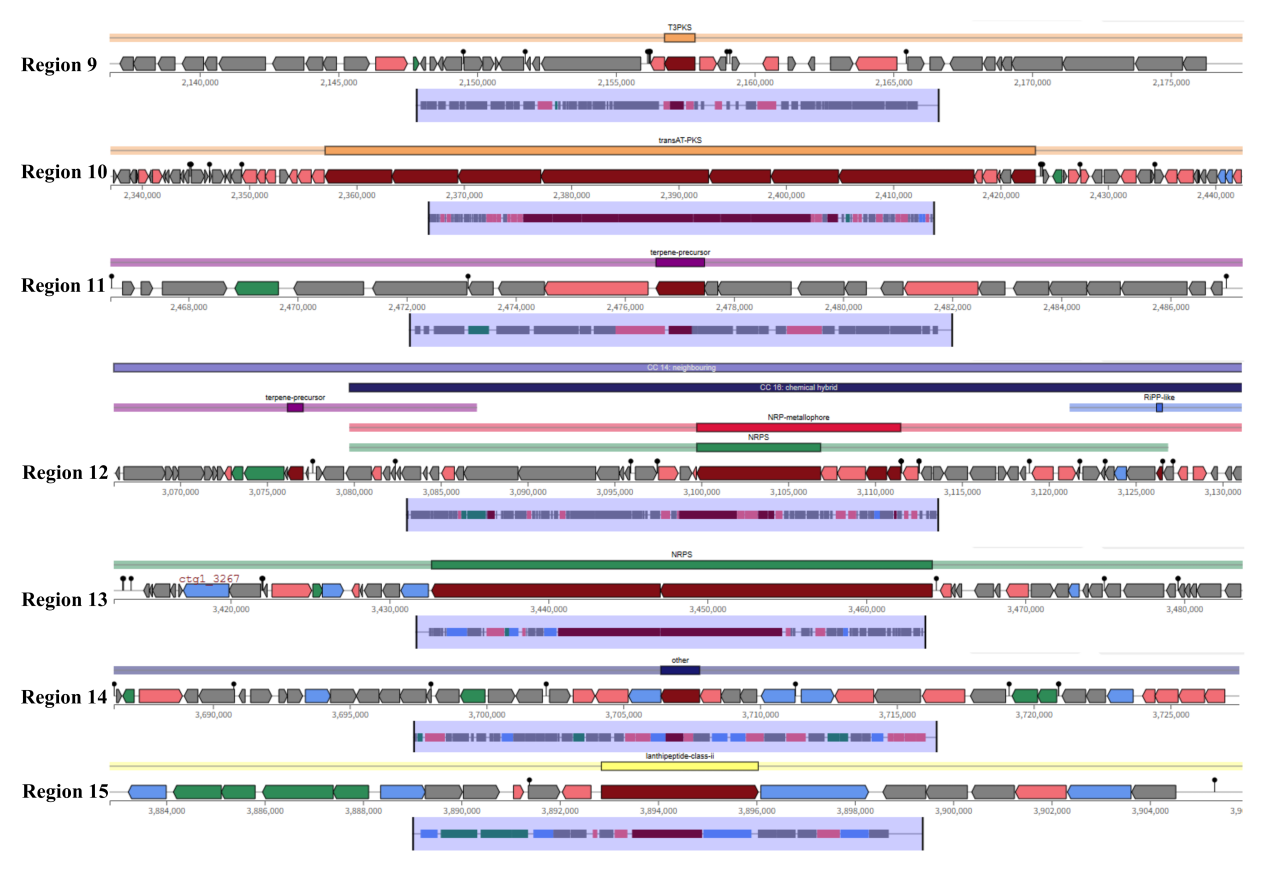
**

**Figure S6.** Predicted results of secondary metabolite biosynthesis gene clusters in the *Bacillus velezensis YNK-FB0059* genome.


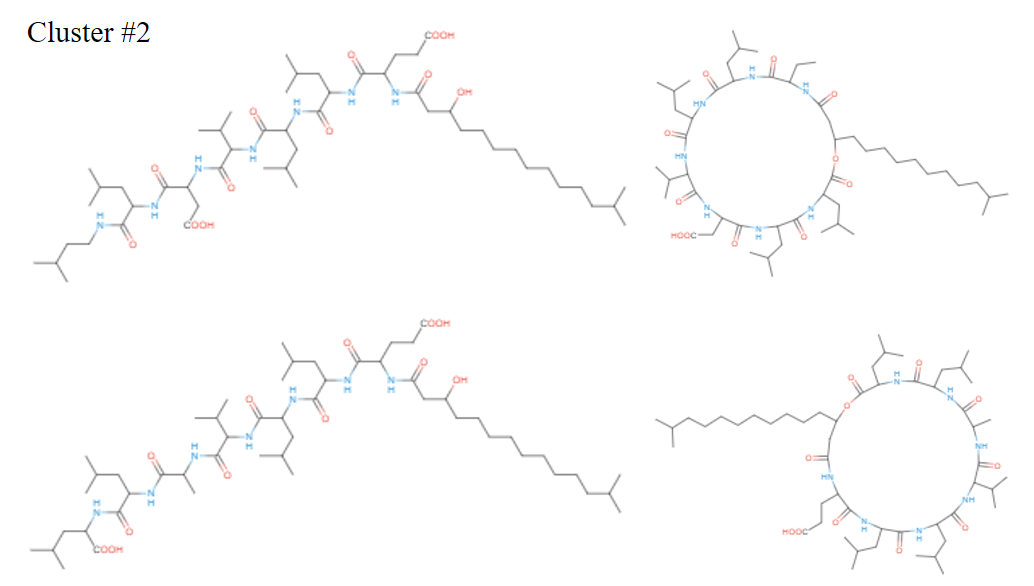

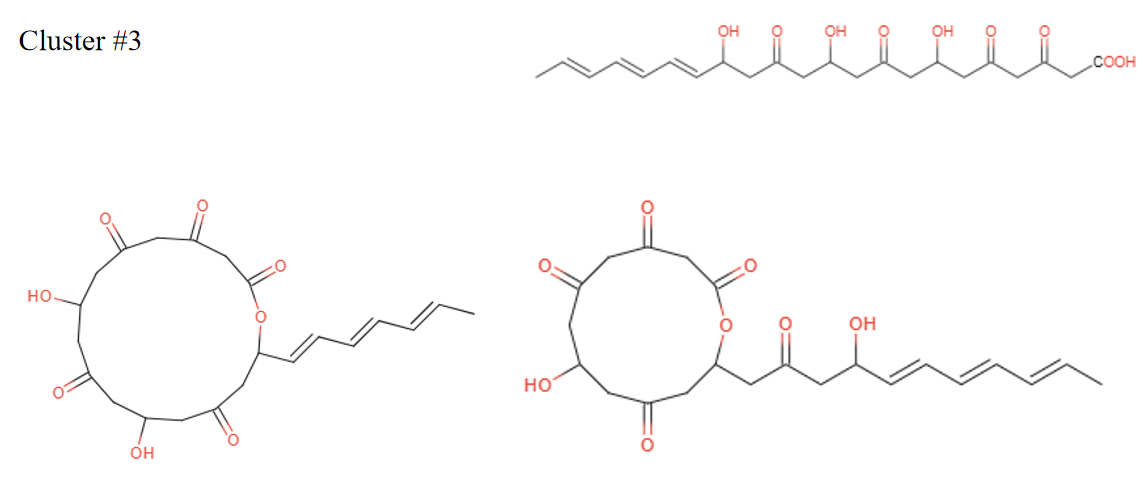

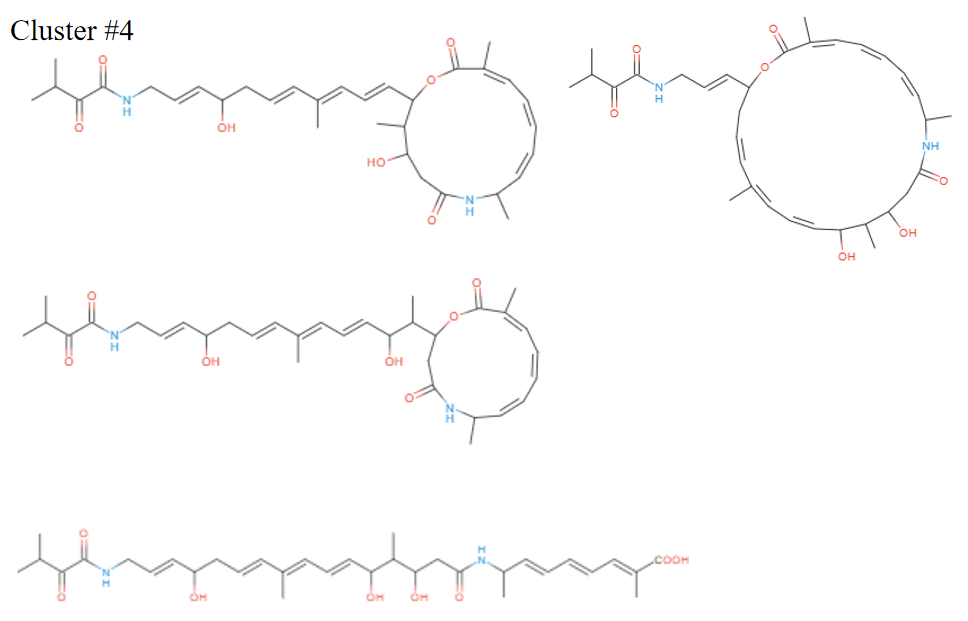

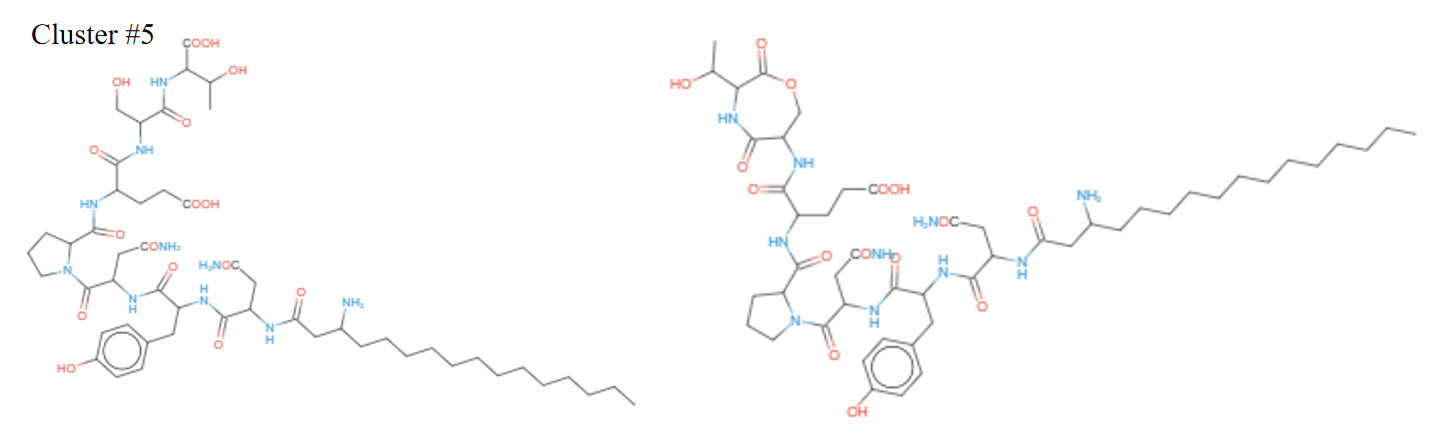

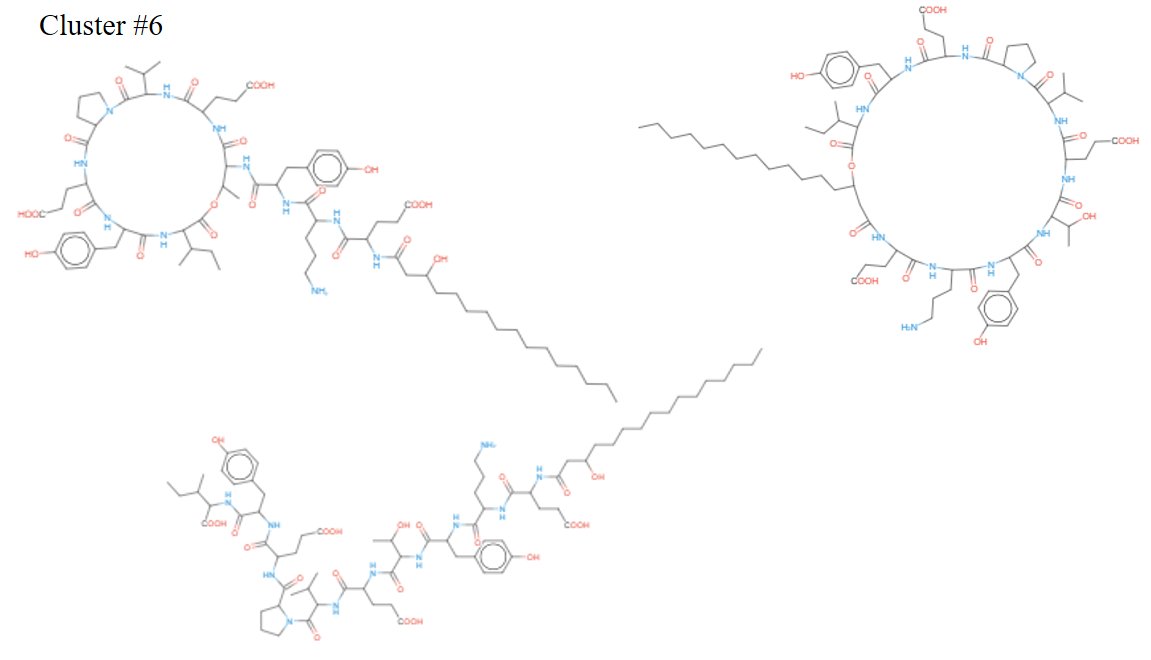

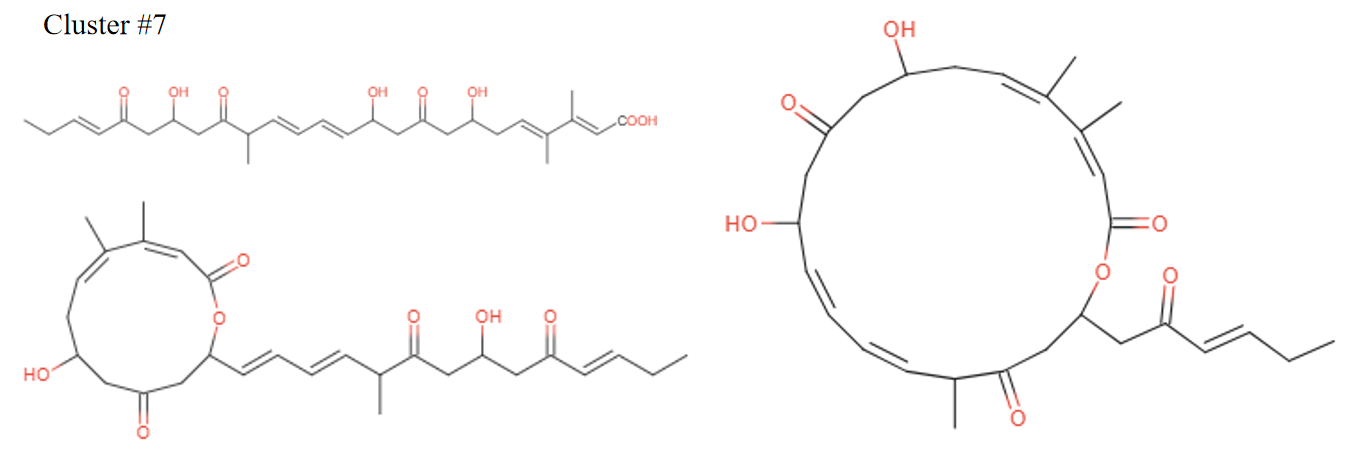

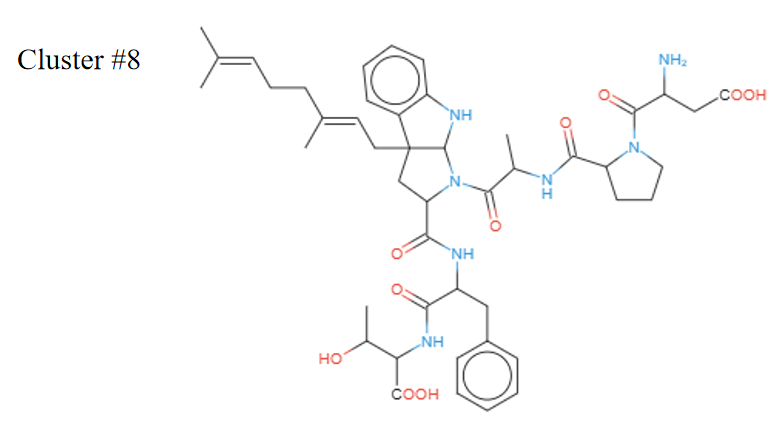


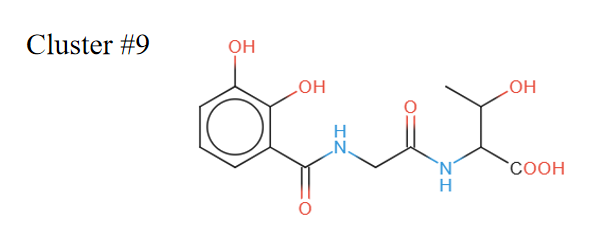

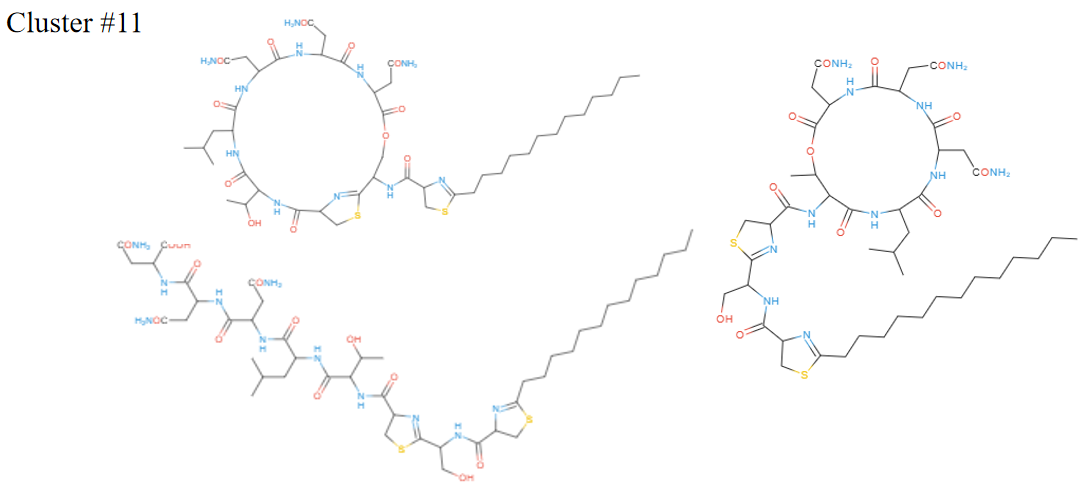

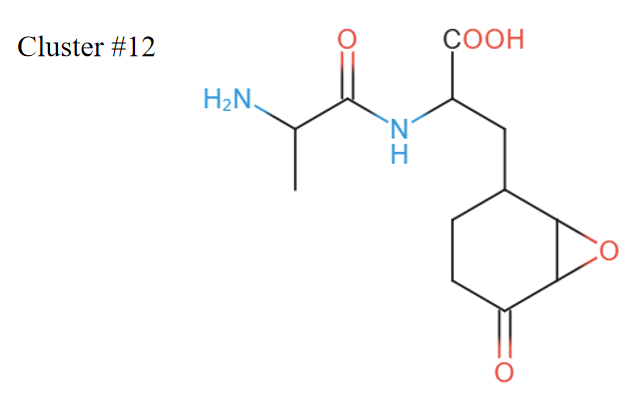

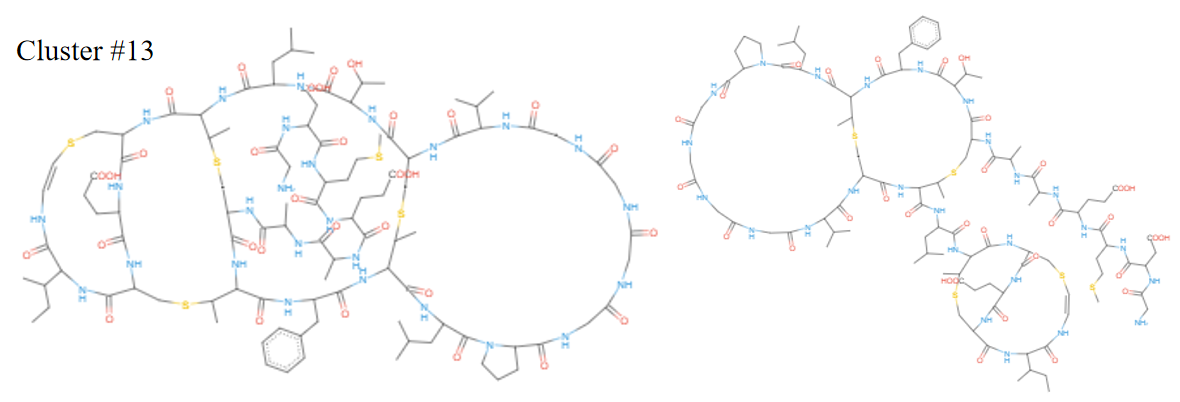


**Fig S7.** Predicted structures of secondary metabolites by Bacillus velezensis YNK-FB0059 using PRISM


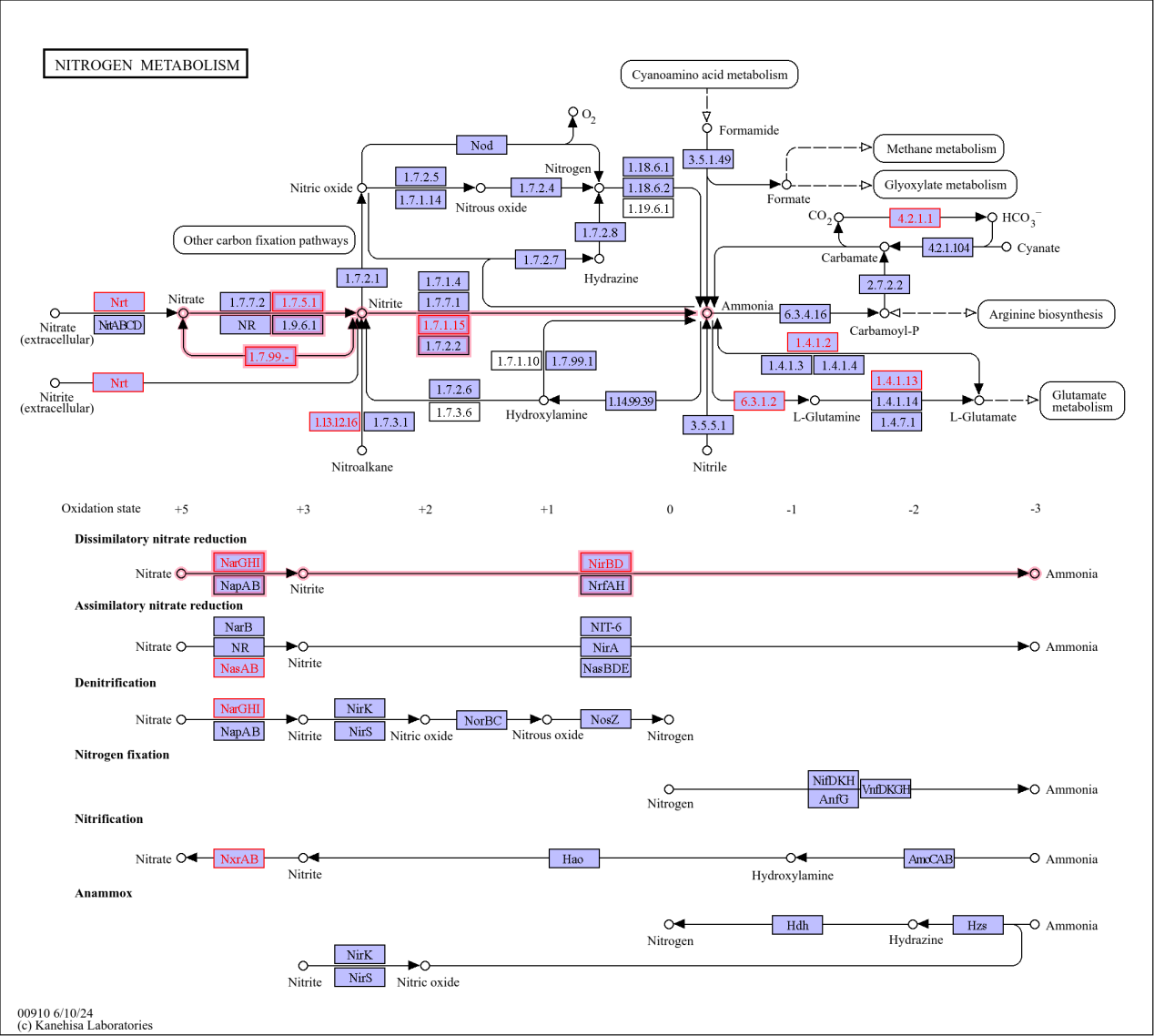


**Figure S8.** Nitrogen metabolism.


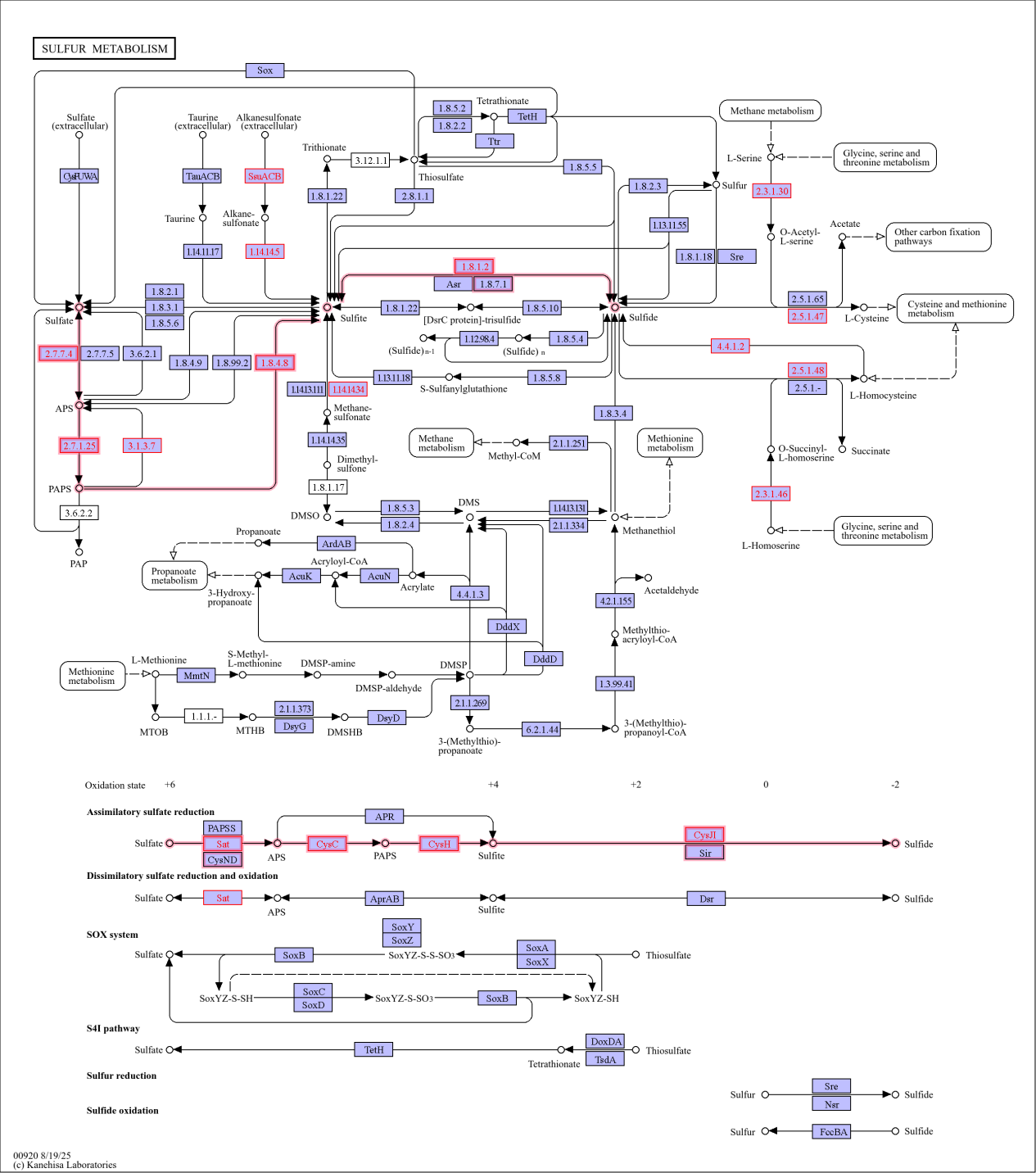


**Figure S9.** Sulfur metabolism.


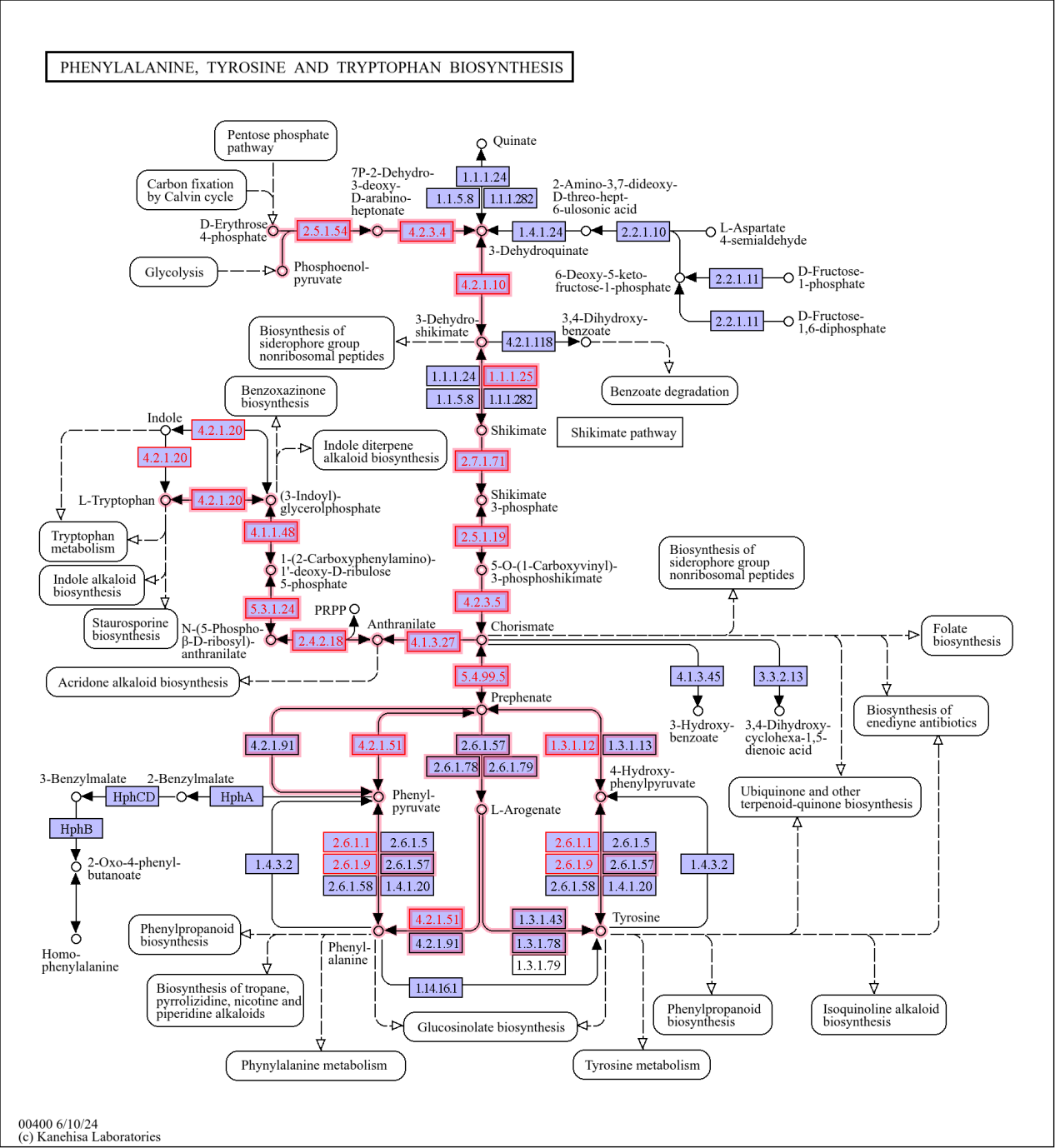


**Figure S10.** Phenylalanine, tyrosine and tryptophan biosynthesis.

Table S1. Mining of Plant Growth-Promoting Related Genes

| Gene ID | KO ID | KO Name | KO Description | |
| --- | --- | --- | --- | --- |
| gene0299 | K09815 | znuA | zinc transport system substrate-binding protein | Genes Related to Nutrient Transport |
| gene0300 | K09817 | znuC | zinc transport system ATP-binding protein [EC:7.2.2.20] |  |
| gene0301 | K09816 | znuB | zinc transport system permease protein |  |
| gene0310 | K02000 | proV | glycine betaine/proline transport system ATP-binding protein [EC:7.6.2.9] |  |
| gene0311 | K02001 | proW | glycine betaine/proline transport system permease protein |  |
| gene0312 | K02002 | proX | glycine betaine/proline transport system substrate-binding protein |  |
| gene0360 | K10010 | tcyC | L-cystine transport system ATP-binding protein [EC:7.4.2.1] |  |
| gene0361 | K10009 | tcyB | L-cystine transport system permease protein |  |
| gene0362 | K02424 | fliY | L-cystine transport system substrate-binding protein |  |
| gene0376 | K16961 | yxeM | putative S-methylcysteine transport system substrate-binding protein |  |
| gene0377 | K16962 | yxeN | putative S-methylcysteine transport system permease protein |  |
| gene0378 | K16963 | yxeO | putative S-methylcysteine transport system ATP-binding protein |  |
| gene1003 | K02073 | metQ | D-methionine transport system substrate-binding protein |  |
| gene1121 | K03523 | bioY | biotin transport system substrate-specific component |  |
| gene1213 | K15580 | oppA | oligopeptide transport system substrate-binding protein |  |
| gene1214 | K15581 | oppB | oligopeptide transport system permease protein |  |
| gene1215 | K15582 | oppC | oligopeptide transport system permease protein |  |
| gene1216 | K15583 | oppD | oligopeptide transport system ATP-binding protein |  |
| gene1217 | K10823 | oppF | oligopeptide transport system ATP-binding protein |  |
| gene1352 | K16200 | dppB1 | dipeptide transport system permease protein |  |
| gene1353 | K16201 | dppC | dipeptide transport system permease protein |  |
| gene1354 | K16202 | dppD | dipeptide transport system ATP-binding protein |  |
| gene1355 | K16199 | dppE | dipeptide transport system substrate-binding protein |  |
| gene2350 | K23060 | artR | arginine/lysine/histidine transport system ATP-binding protein [EC:7.4.2.1] |  |
| gene2351 | K17077 | artQ | arginine/lysine/histidine transport system permease protein |  |
| gene2352 | K23059 | artP | arginine/lysine/histidine transporter system substrate-binding protein |  |
| gene2450 | K02036 | pstB | phosphate transport system ATP-binding protein [EC:7.3.2.1] |  |
| gene2451 | K02036 | pstB | phosphate transport system ATP-binding protein [EC:7.3.2.1] |  |
| gene2452 | K02038 | pstA | phosphate transport system permease protein |  |
| gene2453 | K02037 | pstC | phosphate transport system permease protein |  |
| gene2454 | K02040 | pstS | phosphate transport system substrate-binding protein |  |
| gene2582 | K10041 | glnQ | aspartate/glutamate/glutamine transport system ATP-binding protein [EC:7.4.2.1] |  |
| gene2583 | K10039 | glnH | aspartate/glutamate/glutamine transport system substrate-binding protein |  |
| gene2584 | K10040 | glnP | aspartate/glutamate/glutamine transport system permease protein |  |
| gene2585 | K10040 | glnP | aspartate/glutamate/glutamine transport system permease protein |  |
| gene2718 | K17236 | araQ | arabinooligosaccharide transport system permease protein |  |
| gene2719 | K17235 | araP | arabinooligosaccharide transport system permease protein |  |
| gene2720 | K17234 | araN | arabinooligosaccharide transport system substrate-binding protein |  |
| gene2862 | K10117 | msmE | raffinose/stachyose/melibiose transport system substrate-binding protein |  |
| gene2863 | K10118 | msmF | raffinose/stachyose/melibiose transport system permease protein |  |
| gene2864 | K10119 | msmG | raffinose/stachyose/melibiose transport system permease protein |  |
| gene2913 | K11709 | troD | manganese/zinc/iron transport system permease protein |  |
| gene2914 | K11708 | troC | manganese/zinc/iron transport system permease protein |  |
| gene2915 | K11710 | troB | manganese/zinc/iron transport system ATP- binding protein [EC:7.2.2.5] |  |
| gene2916 | K11707 | troA | manganese/zinc/iron transport system substrate-binding protein |  |
| gene3102 | K10112 | msmX | multiple sugar transport system ATP-binding protein [EC:7.5.2.-] |  |
| gene3126 | K02073 | metQ | D-methionine transport system substrate-binding protein |  |
| gene3127 | K02072 | metI | D-methionine transport system permease protein |  |
| gene3128 | K02071 | metN | D-methionine transport system ATP-binding protein |  |
| gene3192 | K02020 | modA | molybdate transport system substrate-binding protein |  |
| gene3193 | K02018 | modB | molybdate transport system permease protein |  |
| gene3218 | K16960 | tcyN | L-cystine transport system ATP-binding protein [EC:7.4.2.1] |  |
| gene3219 | K16959 | tcyM | L-cystine transport system permease protein |  |
| gene3220 | K16958 | tcyL | L-cystine transport system permease protein |  |
| gene3221 | K16957 | tcyK | L-cystine transport system substrate-binding protein |  |
| gene3222 | K16956 | tcyJ | L-cystine transport system substrate-binding protein |  |
| gene3241 | K05846 | opuBD | osmoprotectant transport system permease protein |  |
| gene3242 | K05845 | opuC | osmoprotectant transport system substrate-binding protein |  |
| gene3243 | K05846 | opuBD | osmoprotectant transport system permease protein |  |
| gene3244 | K05847 | opuA | osmoprotectant transport system ATP-binding protein [EC:7.6.2.9] |  |
| gene3246 | K05846 | opuBD | osmoprotectant transport system permease protein |  |
| gene3247 | K05845 | opuC | osmoprotectant transport system substrate-binding protein |  |
| gene3248 | K05846 | opuBD | osmoprotectant transport system permease protein |  |
| gene3249 | K05847 | opuA | osmoprotectant transport system ATP-binding protein [EC:7.6.2.9] |  |
| gene3319 | K15772 | ganQ | arabinogalactan oligomer / maltooligosaccharide transport system permease protein |  |
| gene3320 | K15771 | ganP | arabinogalactan oligomer / maltooligosaccharide transport system permease protein |  |
| gene3321 | K15770 | cycB | arabinogalactan oligomer / maltooligosaccharide transport system substrate-binding protein |  |
| gene3458 | K10441 | rbsA | ribose transport system ATP-binding protein [EC:7.5.2.7] |  |
| gene3459 | K10440 | rbsC | ribose transport system permease protein |  |
| gene3460 | K10439 | rbsB | ribose transport system substrate-binding protein |  |
| gene3752 | K10112 | msmX | multiple sugar transport system ATP-binding protein [EC:7.5.2.-] |  |
| gene3051 | K03523 | bioY | biotin transport system substrate-specific component |  |
| gene0338 | K00363 | nirD | nitrite reductase (NADH) small subunit [EC:1.7.1.15] | Genes Related to Nitrogen Metabolism |
| gene0339 | K00362 | nirB | nitrite reductase (NADH) large subunit [EC:1.7.1.15] |  |
| gene0340 | K00372 | nasA | assimilatory nitrate reductase catalytic subunit [EC:1.7.99.-] |  |
| gene0341 | K00362 | nirB | nitrite reductase (NADH) large subunit [EC:1.7.1.15] |  |
| gene0342 | K02575 | narK | MFS transporter, NNP family, nitrate/nitrite transporter |  |
| gene1803 | K01915 | glnA | glutamine synthetase [EC:6.3.1.2] |  |
| gene1943 | K00266 | gltD | glutamate synthase (NADPH) small chain [EC:1.4.1.13] |  |
| gene1944 | K00265 | gltB | glutamate synthase (NADPH) large chain [EC:1.4.1.13] |  |
| gene2234 | K00260 | gudB | glutamate dehydrogenase [EC:1.4.1.2] |  |
| gene3590 | K00374 | narI | nitrate reductase gamma subunit [EC:1.7.5.1 1.7.99.-] |  |
| gene3592 | K00371 | narH | nitrate reductase / nitrite oxidoreductase, beta subunit [EC:1.7.5.1 1.7.99.-] |  |
| gene3593 | K00370 | narG | nitrate reductase / nitrite oxidoreductase, alpha subunit [EC:1.7.5.1 1.7.99.-] |  |
| gene3597 | K02575 | narK | MFS transporter, NNP family, nitrate/nitrite transporter |  |
| gene3647 | K00260 | gudB | glutamate dehydrogenase [EC:1.4.1.2] |  |
| gene0087 | K01738 | cysK | cysteine synthase [EC:2.5.1.47] | Genes Related to Sulfur Metabolism |
| gene0122 | K00640 | cysE | serine O-acetyltransferase [EC:2.3.1.30] |  |
| gene0977 | K04091 | ssuD | alkanesulfonate monooxygenase [EC:1.14.14.5 1.14.14.34] |  |
| gene1265 | K01739 | metB | cystathionine gamma-synthase [EC:2.5.1.48] |  |
| gene1612 | K00390 | cysH | phosphoadenosine phosphosulfate reductase [EC:1.8.4.8 1.8.4.10] |  |
| gene1614 | K00958 | sat | sulfate adenylyltransferase [EC:2.7.7.4] |  |
| gene1615 | K00860 | cysC | adenylylsulfate kinase [EC:2.7.1.25] |  |
| gene2070 | K00651 | metA | homoserine O-succinyltransferase/O-acetyltransferase [EC:2.3.1.46 2.3.1.31] |  |
| gene2564 | K17217 | mccB | cystathionine gamma-lyase / homocysteine desulfhydrase [EC:4.4.1.1 4.4.1.2] |  |
| gene2850 | K01738 | cysK | cysteine synthase [EC:2.5.1.47] |  |
| gene3202 | K00381 | cysI | sulfite reductase (NADPH) hemoprotein beta-component [EC:1.8.1.2] |  |
| gene3203 | K00380 | cysJ | sulfite reductase (NADPH) flavoprotein alpha-component [EC:1.8.1.2] |  |
| gene2201 | K01695 | trpA | tryptophan synthase alpha chain [EC:4.2.1.20] | Genes Related to Auxin Precursor Synthesis (Tryptophan Biosynthesis) |
| gene2202 | K01696 | trpB | tryptophan synthase beta chain [EC:4.2.1.20] |  |
| gene2203 | K01817 | trpF | phosphoribosylanthranilate isomerase [EC:5.3.1.24] |  |
| gene2204 | K01609 | trpC | indole-3-glycerol phosphate synthase [EC:4.1.1.48] |  |
| gene2205 | K00766 | trpD | anthranilate phosphoribosyltransferase [EC:2.4.2.18] |  |
| gene2206 | K01657 | trpE | anthranilate synthase component I [EC:4.1.3.27] |  |
| gene0972 | K03781 | katE | catalase [EC:1.11.1.6] | Genes Related to Antioxidant and Stress Tolerance |
| gene3742 | K03781 | katE | catalase [EC:1.11.1.6] |  |
| gene3767 | K03781 | katE | catalase [EC:1.11.1.6] |  |
| gene2908 | K01673 | cynT | carbonic anhydrase [EC:4.2.1.1] | Other Related Genes |
| gene3327 | K01673 | cynT | carbonic anhydrase [EC:4.2.1.1] |  |
| gene0727 | K00812 | aspB | aspartate aminotransferase [EC:2.6.1.1] |  |
| gene1045 | K11358 | yhdR | aspartate aminotransferase [EC:2.6.1.1] |  |
| gene2174 | K00812 | aspB | aspartate aminotransferase [EC:2.6.1.1] |  |
| gene2200 | K00817 | hisC | histidinol-phosphate aminotransferase [EC:2.6.1.9] |  |

Table S2. Mining of Plant Immunity-Enhancing Related Genes

| Gene ID | KO ID | KO Name | KO Description | |
| --- | --- | --- | --- | --- |
| gene3397 | K02406 | fliC | flagellin | Microbe-Associated Molecular Patterns (MAMPs) and Elicitor Synthesis |
| gene1421 | K02556 | motA | chemotaxis protein MotA |  |
| gene2826 | K02556 | motA | chemotaxis protein MotA |  |
| gene1420 | K02557 | motB | chemotaxis protein MotB |  |
| gene2825 | K02557 | motB | chemotaxis protein MotB |  |
| gene1687 | K03413 | cheY | chemotaxis protein CheY |  |
| gene1859 | K03413 | cheY | chemotaxis protein CheY |  |
| gene1697 | K03407 | cheA | sensor kinase CheA [EC:2.7.13.3] |  |
| gene1696 | K03412 | cheB | protein-glutamate methylesterase [EC:3.1.1.61] |  |
| gene2210 | K00575 | cheR | methyltransferase CheR [EC:2.1.1.80] |  |
| gene1447 | K03406 | mcp | methyl-accepting chemotaxis protein |  |
| gene0353 | K15654 | licA | surfactin/lichenysin synthetase A |  |
| gene0354 | K15655 | licB | surfactin/lichenysin synthetase B |  |
| gene0355 | K15656 | licC | surfactin/lichenysin synthetase C |  |
| gene0356 | K15657 | srfATE | external thioesterase TEII |  |
| gene3236 | K20487 | nisK | lantibiotic biosynthesis sensor kinase [EC:2.7.13.3] |  |
| gene3237 | K20488 | nisR | lantibiotic biosynthesis response regulator |  |
| gene3238 | K20492 | nisG | lantibiotic transport permease |  |
| gene3239 | K20491 | nisE | lantibiotic transport permease |  |
| gene3240 | K20490 | nisF | lantibiotic transport ATP-binding protein |  |
| gene0912 | K14205 | mprF | phosphatidylglycerol lysyltransferase [EC:2.3.2.3] | Cell Wall Modification and Immune Evasion/Activation |
| gene3719 | K03367 | dltA | D-alanine--poly(phosphoribitol) ligase [EC:6.1.1.13] |  |
| gene3720 | K03739 | dltB | D-alanine export membrane protein |  |
| gene3721 | K14188 | dltC | D-alanine--poly(phosphoribitol) ligase [EC:6.1.1.13] |  |
| gene3722 | K03740 | dltD | D-alanine transfer protein |  |
| gene2753 | K07636 | phoR | phosphate regulon sensor kinase [EC:2.7.13.3] | Two-Component Systems (Environmental Sensing and Resistance Regulation) |
| gene2754 | K07658 | phoP | alkaline phosphatase synthesis response regulator |  |
| gene2875 | K11629 | bceS | bacitracin resistance sensor kinase [EC:2.7.13.3] |  |
| gene2876 | K11630 | bceR | bacitracin resistance response regulator |  |
| gene3162 | K11618 | liaR | response regulator LiaR |  |
| gene3163 | K11617 | liaS | sensor histidine kinase LiaS [EC:2.7.13.3] |  |
| gene3410 | K07692 | degU | response regulator DegU |  |
| gene3411 | K07777 | degS | sensor histidine kinase DegS [EC:2.7.13.3] |  |
| gene1892 | K01179 | - | endoglucanase [EC:3.2.1.4] | Hydrolases and Cell Wall-Degrading Enzymes (Releasing Elicitors) |
| gene0826 | K01728 | pel | pectate lyase [EC:4.2.2.2] |  |
| gene2019 | K01201 | srfJ | glucosylceramidase [EC:3.2.1.45] |  |
| gene3436 | K23989 | lytD | mannosyl-glycoprotein endo-beta-N-acetylglucosaminidase [EC:3.2.1.96] |  |
| gene3437 | K23989 | lytD | mannosyl-glycoprotein endo-beta-N-acetylglucosaminidase [EC:3.2.1.96] |  |
| gene2910 | K00425 | cydA | cytochrome bd ubiquinol oxidase I [EC:7.1.1.7] | Antioxidant and Stress Response |
| gene2911 | K00426 | cydB | cytochrome bd ubiquinol oxidase II [EC:7.1.1.7] |  |
| gene3749 | K00425 | cydA | cytochrome bd ubiquinol oxidase I [EC:7.1.1.7] |  |
| gene3748 | K00426 | cydB | cytochrome bd ubiquinol oxidase II [EC:7.1.1.7] |  |
| gene2906 | K07173 | luxS | S-ribosylhomocysteine lyase [EC:4.4.1.21] | Other Potential Immune Modulators |
| gene1953 | K11216 | lsrK | autoinducer-2 kinase [EC:2.7.1.189] |  |
| gene3916 | K00692 | sacB | levansucrase [EC:2.4.1.10] |  |
| gene3917 | K01212 | sacC | levanase [EC:3.2.1.65] |  |
